# Micro/mesoporous LTL derived materials for catalytic transfer hydrogenation and acid reactions of bio-based levulinic acid and furanics

**DOI:** 10.3389/fchem.2022.1006981

**Published:** 2022-09-29

**Authors:** Margarida M. Antunes, Andreia F. Silva, Auguste Fernandes, Filipa Ribeiro, Patrícia Neves, Martyn Pillinger, Anabela A. Valente

**Affiliations:** ^1^ Department of Chemistry, CICECO—Aveiro Institute of Materials, University of Aveiro, Aveiro, Portugal; ^2^ Centro de Química Estrutural, Instituto Superior Técnico, Universidade de Lisboa, Lisboa, Portugal

**Keywords:** heterogeneous catalysis, vegetable biomass, zeolite LTL, hafnium, levulinic acid, γ-valerolactone, furanics

## Abstract

The biomass-derived platform chemicals furfural and 5-(hydroxymethyl)furfural (HMF) may be converted to α-angelica lactone (AnL) and levulinic acid (LA). Presently, LA (synthesized from carbohydrates) has several multinational market players. Attractive biobased oxygenated fuel additives, solvents, *etc.,* may be produced from AnL and LA via acid and reduction chemistry, namely alkyl levulinates and γ-valerolactone (GVL). In this work, hierarchical hafnium-containing multifunctional Linde type L (LTL) related zeotypes were prepared via top-down strategies, for the chemical valorization of LA, AnL and HMF via integrated catalytic transfer hydrogenation (CTH) and acid reactions in alcohol medium. This is the first report of CTH applications (in general) of LTL related materials. The influence of the post-synthesis treatments/conditions (desilication, dealumination, solid-state impregnation of Hf or Zr) on the material properties and catalytic performances was studied. AnL and LA were converted to 2-butyl levulinate (2BL) and GVL in high total yields of up to *ca.* 100%, at 200°C, and GVL/2BL molar ratios up to 10. HMF conversion gave mainly the furanic ethers 5-(*sec*-butoxymethyl)furfural and 2,5-bis(*sec*-butoxymethyl)furan (up to 63% total yield, in 2-butanol at 200°C/24 h). Mechanistic, reaction kinetics and material characterization studies indicated that the catalytic results depend on a complex interplay of different factors (material properties, type of substrate). The recovered-reused solids performed steadily.

## 1 Introduction

Global warming and energy security issues related to the intense use of fossil fuels may be alleviated by the alternative use of widespread renewable sources and waste/surpluses to produce bioenergy and bioproducts, thereby avoiding greenhouse gas emissions, environmental pollution, and waste management issues (Roy et al., 2021; Koul et al., 2022). Vegetable biomass existing in terrestrial and aquatic areas constitutes an abundant source of carbohydrates that are partly disposed of as agricultural surpluses/residues, municipal solid waste, industrial (*e.g.*, biorefineries) waste, sewage sludge, etc. (Cheng and Brewer, 2021; Koul et al., 2022; Narisetty et al., 2022). Carbohydrates may be chemically valorized into useful bioproducts for several industrial sectors. The most important selective catalytic routes of carbohydrates to bioproducts include the acid-catalyzed hydrolysis and dehydration reactions under relatively mild conditions, which lead to the bio-based furanic aldehyde platform chemicals 5-(hydroxymethyl)furfural (HMF) and furfural (FUR) (Liguori et al., 2015; Dutta, 2021; Fact, 2022).

**SCHEME 1:**
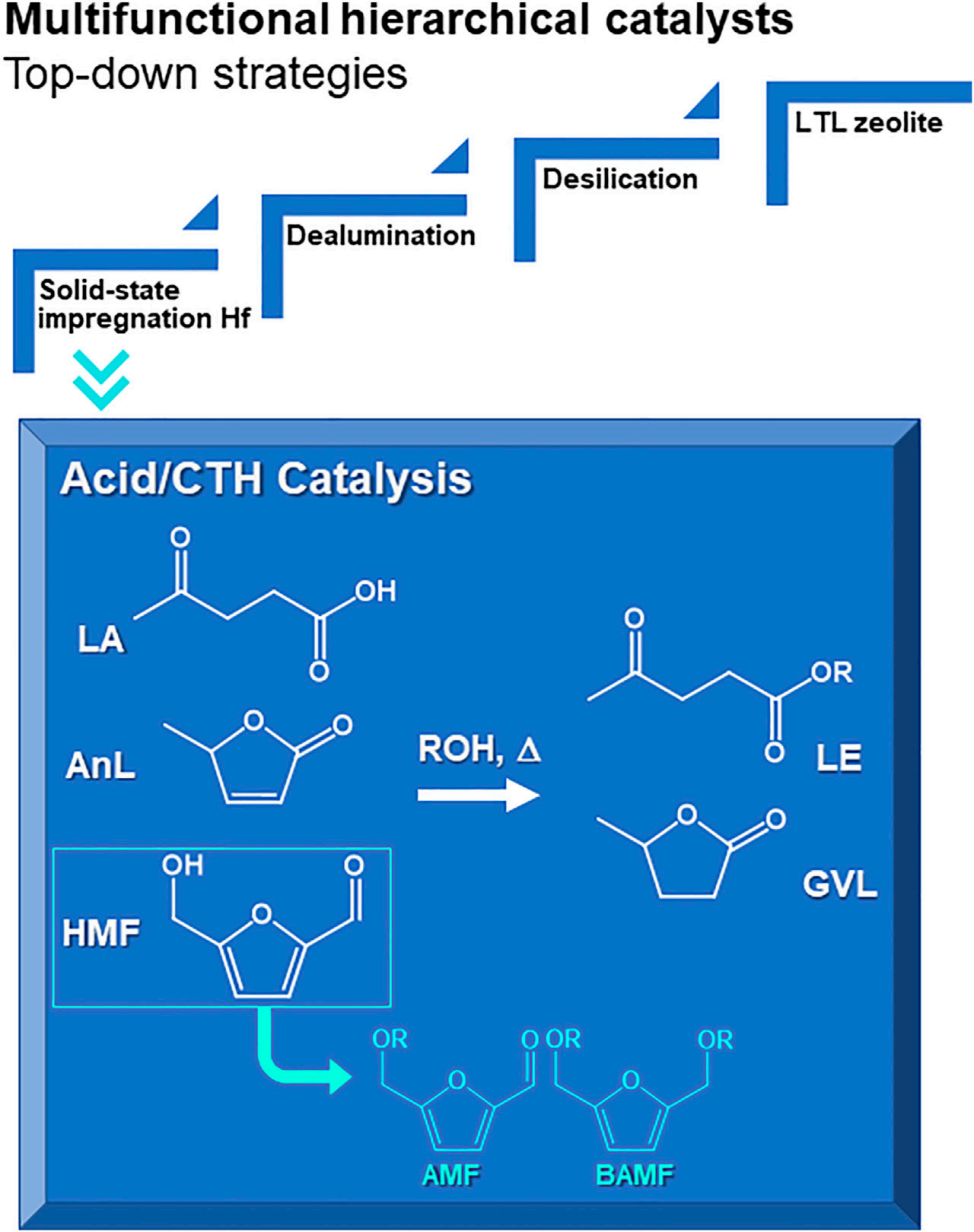
Hierarchical multifunctional LTL related materials prepared via top-down strategies for integrated catalytic transfer hydrogenation (CTH) and acid reactions of levulinic acid (LA), α-angelica lactone (AnL) and 5-(hydroxymethyl)furfural (HMF) to useful bioproducts such as alkyl levulinates (LE) and γ-valerolactone (GVL), or (from HMF) 5-(alkoxymethyl)furfural (AMF) and 2,5-bis(alkoxymethyl)furan (BAMF).

HMF may be selectively converted to oxygenated fuel additives in alcohol media, namely 5-(alkoxymethyl)furfural (AMF) and 2,5-bis(alkoxymethyl)furan (BAMF) via etherification or reduction/etherification, respectively (Scheme 1) (Poullikkas, 2017; Kong et al., 2020; Hou et al., 2021). AMFs possess relatively high energy density, low toxicity and high stability (Li et al., 2016b; Kong et al., 2018; Hou et al., 2021; Liu et al., 2022). On the other hand, BAMFs may possess higher energy density, cetane number, miscibility and stability than AMFs, making them even more attractive fuel additives (Hu et al., 2018, 2020); *e.g.*, BAMFs synthesized using *n*-butanol (which may be renewable and synthesized via fermentation of hexoses (Cho et al., 2014)) or secondary and tertiary alcohols (Gruter, 2009).

On the other hand, HMF and FUR may be converted to α-angelica lactone (AnL) and levulinic acid (LA), and AnL may be an intermediate to LA (Al-Shaal et al., 2015; Antonetti et al., 2016; Lima et al., 2018; Dutta and Bhat, 2021). LA was identified as one of the most valuable bioproducts derived from carbohydrates (Werpy and Petersen, 2004), and its market players include Biofine and DuPont (Fitzpatrick, 1995; Grand View Research, 2021). The growing LA market covers several sectors, from pharmaceuticals, agrochemicals, polymers, plasticizers, fuel and fuel additives to flavours, fragrances, cosmetics, and food additives (Morone et al., 2015; Dutta and Bhat, 2021; Di Bucchianico et al., 2022; Mordor Intelligence, 2022).

Both AnL and LA may be converted to alkyl levulinates (LE) and γ-valerolactone (GVL) (Manzer, 2005; Liguori et al., 2015; Zhang, 2016; Lima et al., 2018; Yu et al., 2019; Dutta and Bhat, 2021; Antunes et al., 2022a). LE and GVL are attractive oxygenated fuel additives (Morone et al., 2015); *e.g.*, butyl levulinate blends can improve fuel properties such as conductivity, combustion emission, freezing point (Di Bucchianico et al., 2022). Furthermore, GVL has a growing market as a food flavouring agent and solvent, foreseen up to 2028 (Data Intelo, 2022), and is an interesting intermediate to diverse chemicals and fuels (Yu et al., 2019; Dutta and Bhat, 2021; Sajid et al., 2021).

The conversion of AnL and LA to GVL involves acid and reduction chemistry (Scheme 1). Hence, reducing agents are required, which may be H_2_ (for hydrogenation) or organic H-donors such as secondary alcohols or acids (for catalytic transfer hydrogenation (CTH)) (Dutta et al., 2019; Yu et al., 2019; Yu et al., 2020b, 2020a; Weng et al., 2020; Maumela et al., 2021). High GVL yields were reported for the reaction of LA with H_2_ (mainly produced from fossil fuels) using (expensive) noble metal catalysts (Huang et al., 2020; Bassi et al., 2021; Lu et al., 2022; Vu et al., 2022). However, the process economics and safety may be favoured by using (cheaper) non-noble metal catalysts (Xue et al., 2018; Dutta et al., 2019; Wang et al., 2020) and, on the other hand, organic H-donors instead of operating under high pressure H_2_ conditions, which requires robust, expensive infrastructures (Gilkey and Xu, 2016; Dutta et al., 2019; Nie et al., 2021).

A literature survey indicated that hafnium-containing catalysts may be effective for LA conversion to GVL via CTH chemistry, *i.e.*, not requiring external H_2_ supply and noble metals, specifically: hafnium supported on organic supports such as sulfonated sugar cane bagasse (Huang et al., 2021), carbonized/sulfonated glucose (Jori and Jadhav, 2020), or graphite oxide (Li et al., 2020); hafnium-containing organic-inorganic coordination hybrids (including MOFs) (Xie et al., 2016; Rojas-Buzo et al., 2018; Antunes et al., 2022b); and zeolites (BEA (Antunes et al., 2022a), FAU (Tang et al., 2019)). According to the literature, Hf-containing catalysts (zeolites, hybrids) may perform superiorly to the Zr-analogues in the conversion of LA and LE to GVL (Luo et al., 2014; Tang et al., 2019; Li et al., 2020; Antunes et al., 2022a; 2022b). Of these, the non-carbon-based catalysts may possess longer lifetimes and better resistance against regeneration treatments such as calcination of coke/humins, enhancing productivity. In this sense, zeolites may be promising.

Zeolites are (fully inorganic) crystalline microporous aluminosilicates used in the industry, and are particularly attractive for catalytic and adsorption processes (Čejka et al., 2020). They possess considerable specific surface area, tuneable acidity and selectivity properties (Li et al., 2016b; Mon and Leyva-Pérez, 2021). Different zeolite topologies are commercially available (Kuznetsova et al., 2018), which may be modified via top-down or bottom-up strategies, broadening and/or improving their catalytic applications. For example, hierarchical (micro/mesoporous) zeotype catalysts may be effective for multiple reactions (Serrano et al., 2018). The few literature studies on the conversion of LA to GVL over micro/mesoporous zeotypes led to 22–99% GVL yield (Antunes et al., 2022a): Sn-containing Beta (Antunes et al., 2015; Winoto et al., 2016); Zr-containing Beta (Antunes et al., 2016a, 2016b; Morales et al., 2019; López-Aguado et al., 2020); Hf-USY (Tang et al., 2019) and Hf-AlBeta (Antunes et al., 2022a) which surpassed the performances of Sn- and Zr-counterparts. These promising results motivated us to explore different commercially available zeolite topologies and post-synthesis treatments for GVL production.

Linde type L zeolites consist of a microporous aluminosilicate framework (K_6_Na_3_(H_2_O)_21_[Al_9_Si_27_O_72_] for Si/Al = 3, but higher ratios are possible) with LTL topology, which allows diffusion along 1D 12-membered ring (MR) channels that have *ca*. 0.71 nm × 0.71 nm apertures and cages with *ca.* 1.2 nm diameter (Broach, 2010). The 1D 12 MR channel system is connected by a 3D 8 MR channel system (0.34 nm × 0.56 nm). LTL zeolites possess easily exchangeable cations located inside the 12 MR channels near the 8 MR windows. Although a literature survey indicates that LTL zeolitic catalysts are effective for hydrogenation reactions using externally supplied H_2_ (details discussed in the Supplementary Material), to the best of our knowledge, LTL catalysts were never reported in the literature for CTH reactions, in general.

Few hierarchical LTL type materials were prepared via different top-down strategies (*e.g.*, with or without surfactants) and conditions (leading to enhanced mesopore volume (V_meso_)), partly depending on the catalytic applications: alkane aromatization (V_meso_ increased from 0.079 to 0.44 cm^3^ g^−1^) (Lee and Choi, 2016); Knoevenagel condensation (V_meso_ increased from 0.01 to 0.29 cm^3^ g^−1^) (Nilesh et al., 2016); dealkylation of aromatics (V_meso_ increased from 0.08 to 0.28 cm^3^ g^−1^) (Al-Ani et al., 2019); and hydrogenation of d-xylose to xylitol (V_meso_ increased from 0.010 to 0.14 cm^3^ g^−1^) (Tangale et al., 2019). Al-Ani *et al.* (Al-Ani et al., 2019) proposed that the formation of mesopores in LTL via mixed alkaline plus surfactant treatment was gradual in relation to that for more open pore structures such as the FAU and BEA topologies, allowing some control over the features of the LTL pore system.

In this work, hierarchical multifunctional LTL zeotypes were prepared for integrated CTH and acid reactions of LA, AnL and HMF to useful bioproducts (Scheme 1). The catalysts were prepared from commercial KL zeolite via top-down strategies involving desilication, dealumination and solid-state impregnation (SSI) of hafnium (and zirconium for comparison). This is the first CTH application reported for the LTL family of zeolites. The influence of the post-synthesis treatments (*e.g.*, with or without desilication) and conditions (*e.g.*, acid concentration, hafnium loading) on the material properties and catalytic performances was studied. Mechanistic, reaction kinetics and material characterization studies indicated that the catalytic results depend on a complex interplay of different factors, and the relationships of material properties-catalytic performance may be different for different substrates.

## 2 Materials and methods

All reagents and solvents were obtained from commercial sources and used as received (purities and suppliers are indicated in Supplementary Material). The materials were characterized (details given in the Supplementary Material) by powder X-ray diffraction (PXRD; crystalline structure, relative crystallinity (RC)), scanning electron microscopy (SEM; morphology), elemental mappings (distributions of Hf, Zr, Si, Al) and energy dispersive X-ray spectroscopy (EDS; Si/Al, Si/Hf ratios), nitrogen sorption isotherms (textural properties and pore size distributions), ^27^Al MAS NMR spectroscopy (types of Al sites), attenuated total reflectance Fourier Transform Infrared (ATR FT-IR) spectroscopy (surface chemistry), elemental analysis for carbon (adsorbed carbonaceous matter on the used catalysts), and FTIR of adsorbed pyridine (base probe) at 150 and 350°C (acid properties).

### 2.1 Preparation of the catalysts

Hafnium-containing hierarchical LTL zeotypes and a Zr-counterpart were prepared via post-synthesis partial dealumination using oxalic acid (oxac), desilication (D) using KOH treatments, followed by dealumination using H_2_SO_4_, solid-state impregnation (SSI) of hafnium acetylacetonate used as Hf precursor and finally calcination.

#### 2.1.1 Aluminosilicates

Commercial KL zeolite was mildly dealuminated using 0.08 M aq. oxalic acid (30 ml solution per gram of zeolite) at 80°C for 3 h, with stirring. The solid was separated by filtration, thoroughly washed with hot Milli-Q water until neutral pH, dried overnight at 100°C, and finally calcined at 550°C (1°C min^−1^) in static air for 5 h, giving KL(oxac). Literature studies reported alkaline treatments of LTL type materials, using NaOH or KOH in the concentrations range 0.04–2.8 M (Lee and Choi, 2016; Nilesh et al., 2016; Al-Ani et al., 2019; Tangale et al., 2019). KL(oxac) was subsequently desilicated (D) using 0.2 M aq. KOH (30 ml per gram of zeolite) at 65°C for 30 min, with stirring. The mixture was cooled in an ice bath during 10 min and the solid was separated by filtration, thoroughly washed with hot Milli-Q water until neutral pH, and dried overnight at 100°C, giving D-KL.

D-KL was simultaneously ion-exchanged and dealuminated using H_2_SO_4_. According to the literature, KL may tolerate 0.4 M H_2_SO_4_ treatment for 1 h (Al-Ani et al., 2019); these conditions were applied to D-KL, but led to complete amorphization (Supplementary Material for details, Supplementary Figure S1). Hence, the concentration and time of the H_2_SO_4_ treatment were reduced. Specifically, D-KL was treated with 0.10, 0.15, 0.20 or 0.28 M aq. H_2_SO_4_ (30 ml per gram of zeolite) for 30 min at room temperature, with stirring. The solid was separated by filtration, thoroughly washed with hot Milli-Q water until neutral pH, and dried overnight at 100°C, giving D-HL(x), where x stands for the concentration of H_2_SO_4_ (0.10 ≤ x < 0.28 M).

For comparison, a material denoted HL was prepared by subjecting commercial KL to dealumination with 0.15 M aq. H_2_SO_4_ and then SSI of 0.28 mmol_Hf_ g^−1^ (the two treatments were carried out in a comparable fashion to that described above for the D-HL(x) materials). In a separate experiment, commercial KL was subjected to ion-exchange using 0.15 M aq. NH_4_NO_3_; the resultant material exhibited additional PXRD peaks, suggesting the presence of crystalline contaminations (which was reproducible; please see the Supplementary Material for details, Supplementary Figure S2), and, thus, this material was discarded.

#### 2.1.2 Hf-containing zeotypes and Zr-counterpart

The D-HL(x) precursors were subjected to SSI with Hf(acac)_4_ and subsequent calcination, giving yHf-D-HL(x) where y is the hafnium concentration (in the range 0.28–1.68 mmol_Hf_ g^−1^). The SSI process involved gentle grinding of 1 g of the aluminosilicate support with the desired amount of Hf(acac)_4_ precursor for 30 min, using an agate pestle and mortar. The resultant material was calcined at 550°C (1°C min^−1^) under air flow (20 ml min^−1^) for 6 h. Zeolite 0.28Hf-HL was prepared in a similar fashion, albeit using the support HL and y = 0.28 mmol_Hf_ g^−1^. The Zr-counterpart, namely 0.28Zr-D-HL(0.15), was prepared by the same protocol, albeit using Zr(acac)_4_ as precursor (0.158 g Zr(acac)_4_ per g of D-HL(0.15)).

### 2.2 Catalytic tests

The catalytic reactions were carried out using homemade tubular glass batch reactors (*ca.* 8 cm length, *ca.* 11 mm internal diameter) with a conic-shaped bottom, equipped with a PTFE-coated magnetic stirring bar (Supelco) and a PTFE valve (Normax) for purging. Each reactor was loaded with LA (in a concentration ranging from 0.14 to 0.45 M), catalyst (25.5 g_cat_ L^−1^) and 2BuOH. The reactions of AnL and HMF were carried out using an initial substrate concentration of 0.45 M in 2BuOH. Bulk HfO_2_ was used in an equivalent molar amount of hafnium to that added together with the catalysts 1.40Hf-D-HL(x) to the reactor. Prior to control tests using D-HL(x), these materials were calcined under similar conditions to those described above for the Hf-containing zeotypes.

The loaded reactors were immersed in a thermostatically controlled oil bath heated at 200°C and stirred to 850 rpm to favour uniform temperature distribution and avoid external diffusional limitations. Reaction time was counted from the instant that the reactors were immersed in the oil bath. After a given reaction time, the reactors were cooled to room temperature prior to sampling. Freshly prepared samples were analyzed by HPLC for quantification of HMF or by gas chromatography (GC) for quantification of the reaction products and remaining substrates.

The HPLC analyses were carried out using a Knauer Smartline HPLC Pump 100 and a Shodex SH1011H + 300 mm × 8 mm (i.d.) ion exchange column (Showa Denko America, Inc. New York), coupled to a Knauer Smartline 2520 UV detector (254 nm). The column temperature was set at 50°C, and the mobile phase was 0.005 M aq. H_2_SO_4_ at a flow rate of 0.8 ml min^−1^. The GC analyses were carried out using an Agilent 7820A GC equipped with a capillary column (HP-5, 30 m × 0.320 mm × 0.25 mm; 35–300°C), spit injector (240°C) and a flame ionization detector (320°C), using H_2_ as carrier gas. The quantification of the substrates and reaction products was based on calibration curves using internal standards. Individual experiments were performed for a given reaction time, and the presented results are the mean values of at least two replicates with an error <6%.

The reaction products were identified using a Shimadzu QP2010 ultra-GC-MS (Izasa Scientific, Lisbon, Portugal) equipped with a split injector (240°C), ion source (200°C), interface (300°C), a Zebron ZB-5ms capillary GC column (ZB-5, 30 m × 0.25 μm × 0.25 mm; 35–300°C) and He as carrier gas (supporting databases: Wiley229 and NIST14). The identified products were 2-butyl levulinate (2BL) and GVL from LA; LA, 2BL and GVL from AnL; 5-methylfurfural (5MF), 5-(*sec*-butoxymethyl)furfural (BMF) and 2,5-bis(*sec*-butoxymethyl)furan (BBMF) from HMF.

The conversion (%) of the substrate (Sub) at a reaction time *t* was calculated using the formula, 100 × [(initial molar concentration of Sub)—(molar concentration of Sub at reaction time t)/(initial molar concentration of Sub)], and product (Prod) yield was calculated using formula 100 × [(molar concentration of Prod at time t)/(initial molar concentration of Sub)]. Catalytic activities (mmol g_cat_ h^−1^) were calculated based on the substrate conversion at 5 h reaction.

The used catalysts were separated by centrifugation (10,000 rpm for *ca.* 5 min), thoroughly washed with the reaction solvent (2BuOH) and dried at 85°C overnight. The used catalysts were thermally regenerated at 550°C (heating rate of 1°C min^−1^) for 5 h, under an air flow (20 ml min^−1^). Selected catalysts (1.40Hf-D-HL(0.15), 1.12Hf-D-HL(0.28)) were used for five consecutive 5 h-batch runs (0.45 M LA, 2BuOH, 25.5 g_cat_ L^−1^, 200°C).

Contact tests (CT) consisted of contacting the fresh catalysts 1.40Hf-D-HL(0.15) and 1.12Hf-D-HL(0.28) with 2BuOH at 200°C for 5 h, under the same conditions as those used for a normal catalytic test, but without substrate; then, the solid was separated by centrifugation (10,000 rpm) and the supernatant liquid phase was passed through a 220 μm pore size PTFE membrane; the substrate (LA) was added to this solution (to give an initial concentration of 0.45 M) and left to react for 5 h at 200°C (giving LP-CT); finally, the homogeneous mixture was analyzed by GC and the results were compared to those without catalyst.

## 3 Results and discussion

### 3.1 Characterization of the catalysts

Multifunctional LTL related materials (yHf-D-HL(x)) were prepared from commercial KL (Si/Al = 3) via top-down strategies for the target reactions of LA, AnL and HMF. The strategies consisted of mild dealumination (giving KL(oxac), Si/Al = 5); desilication (D) to introduce mesoporosity (giving D-KL, Si/Al = 3); dealumination using H_2_SO_4_ (giving D-HL(x), Si/Al in the range 5–41); solid-state impregnation (SSI) of hafnium and calcination. The influence of the acid concentration (x in the range 0.10–0.28 M H_2_SO_4_) and Hf loading (y in the range 0.28–1.68 mmol_Hf_ g^−1^) on the material properties was studied. The characterization studies of the zirconium counterpart 0.28Zr-D-HL(0.15) are discussed in the Supplementary Material (Supplementary Figure S3, Supplementary Table S1); the results were comparable to those for 0.28Hf-D-HL(0.15).

**FIGURE 1:**
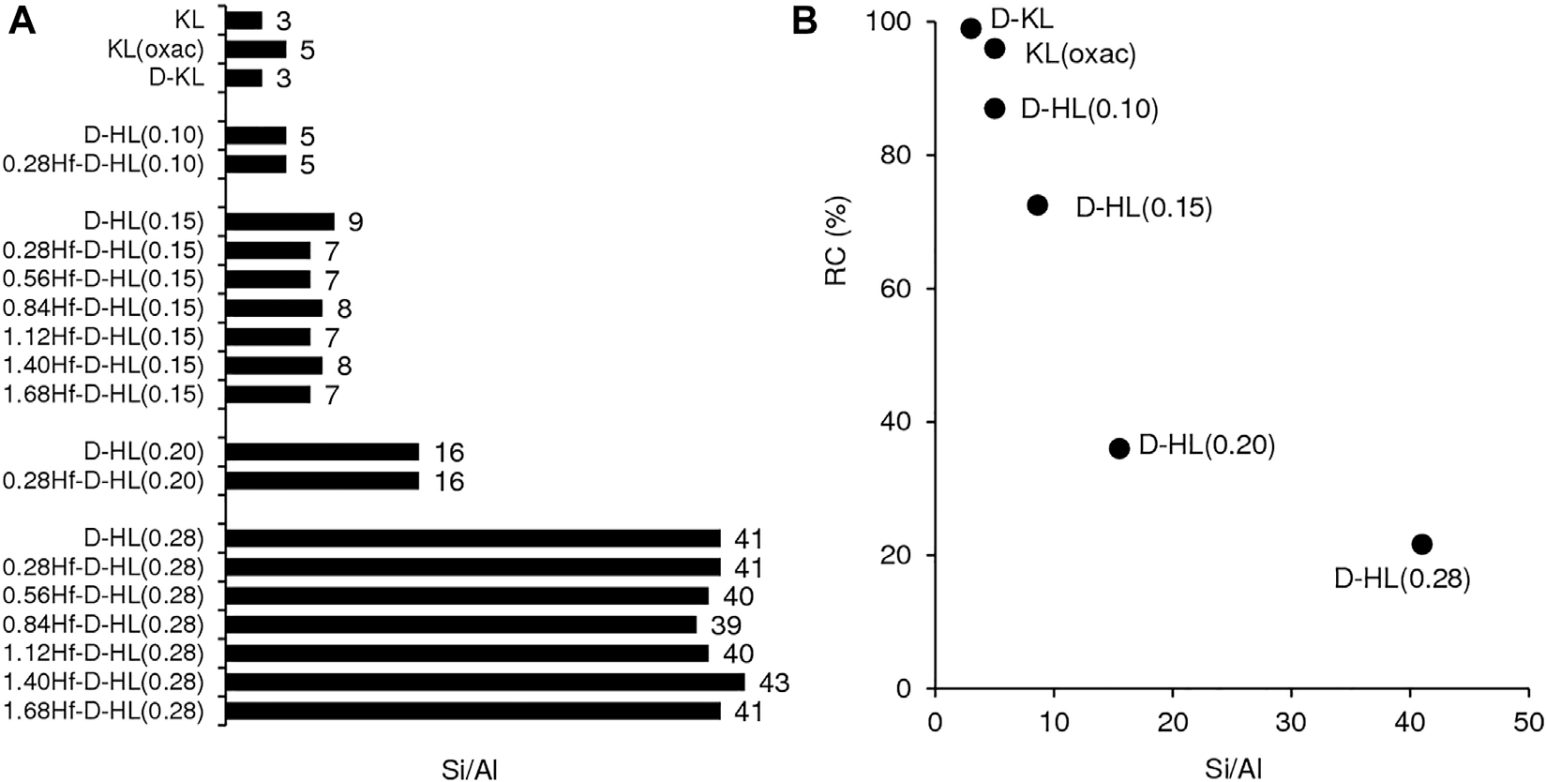
Si/Al ratio of the prepared materials **(A)** and relative crystallinity (RC) as a function of Si/Al **(B)**. The RC values are given in Table S1.

For the materials D-HL(x), increasing x led to increasing Si/Al ratio in the range 5-41, suggesting that partial removal of aluminum occurred (likely forming vacant sites) (Figure 1A, Supplementary Table S1). Although the materials HL (Si/Al = 6) and D-HL(0.15) (Si/Al = 9) were subjected to the same acid treatment using 0.15 M H_2_SO_4_, the latter possessed a higher Si/Al ratio, suggesting that the pretreatments facilitated dealumination. The SSI process did not significantly affect the Si/Al ratio, *i.e.*, each pair of materials D-HL(x) and yHf-D-HL(x) with the same x possessed similar Si/Al ratio. Since no wash/extraction/filtration operations were performed after SSI, the amount of hafnium introduced in the SSI step remained in the solids, giving yHf-D-HL(x) with y in the range 0.28–1.68 mmol_Hf_ g^−1^ (Supplementary Table S1).

**FIGURE 2:**
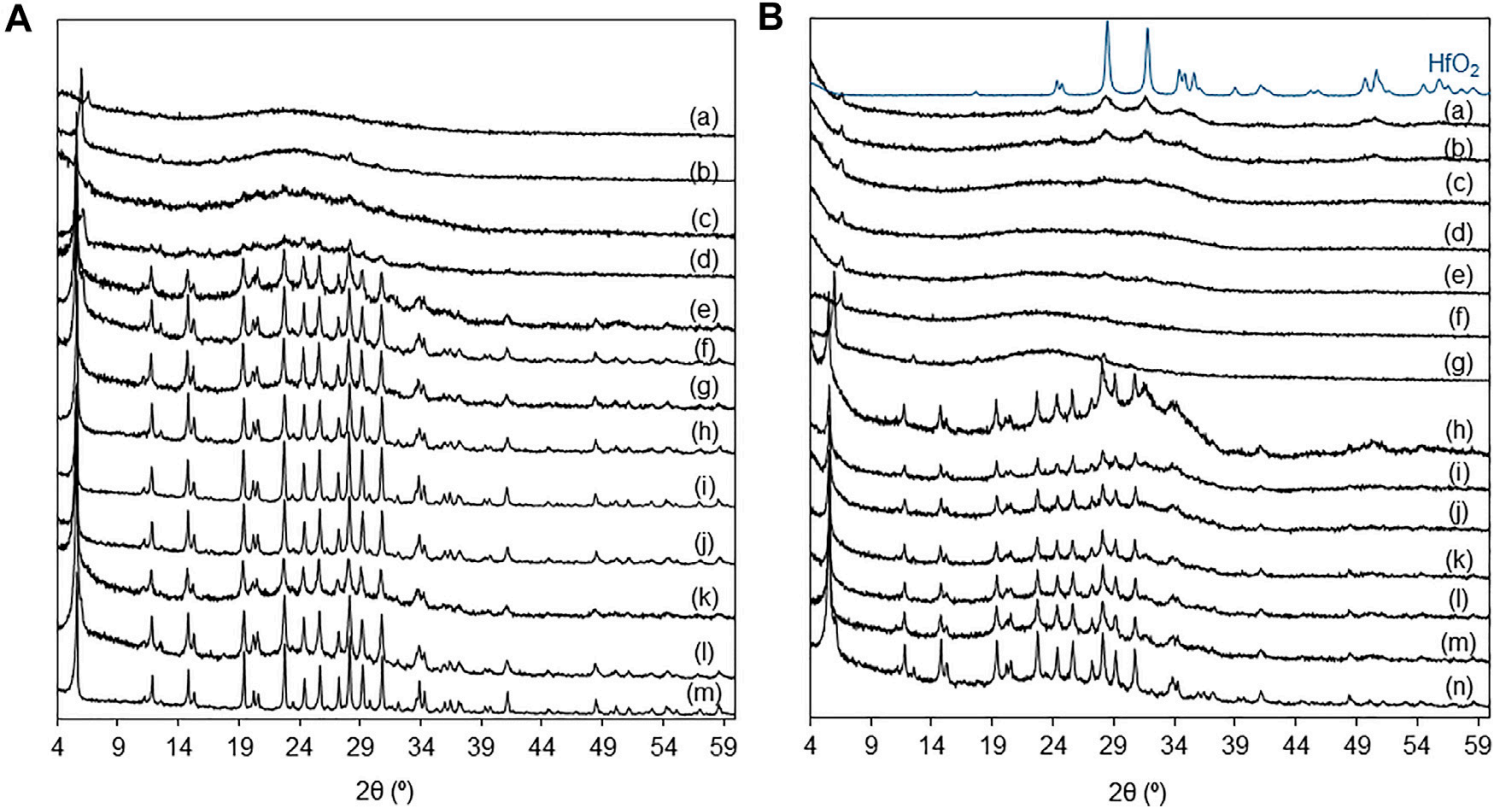
**(A)** PXRD patterns of (a) D-HL(x) with x = 0.28 (b), 0.20 (d), 0.15 (f), 0.10 (h); 0.28Hf-D-HL(x) with x = 0.28 (a), 0.20 (c), 0.15 (e), 0.10 (g); D-KL (i); KL (oxac) (j); 0.28Hf-HL (k); HL (l); KL (m). **(B)** PXRD patterns of yHf-D-HL(0.28) with y = 1.68 (a), 1.40 (b), 1.12 (c), 0.84 (d), 0.56 (e), 0.28 (f); D-HL(0.28) (g); yHf-D-HL(0.15) with y = 1.68 (h), 1.40 (i), 1.12 (j), 0.84 (k), 0.56 (l), 0.28 (m); D-HL(0.15) (n); bulk HfO_2_.

The PXRD patterns of the materials KL, KL(oxac), HL, D-KL, D-HL(x) and yHf-D-HL(x) with x ≤ 0.15 indicated that the LTL topology was preserved (Lee and Choi, 2016; Nilesh et al., 2016; Al-Ani et al., 2019; Tangale et al., 2019), whereas the materials with x = 0.20 and 0.28 were mostly amorphous (Figure 2, Supplementary Table S1). The PXRD patterns of yHf-D-HL(0.15) with y < 1.68 mmol_Hf_ g^−1^ and of yHf-D-HL(0.28) with y < 1.40 mmol_Hf_ g^−1^ did not evidence the presence of other crystalline phases; peaks characteristic of hafnia monoclinic phase include *ca.* 28.6°, 31.8° and 34.7° (ICDD PDF-4+ 2020 reference code no. 04-005–4477) (Figure 2B). These results suggest that these materials possessed somewhat uniform metal/metalloid (Si, Al, Hf) distributions. The remaining materials with higher y exhibited weak reflections at *ca.* 28° and 32° assignable to polymeric hafnium oxide species. For the highest Hf loading of 1.68 mmol_Hf_ g^−1^ (for which formation of hafnium oxide particles may be more critical), the average crystallite size of the supported hafnium oxide particles (considering a spherical geometry) was very small, *ca.* 8–9 nm.

The oxalic acid and desilication treatments of KL did not significantly affect the relative crystallinity (RC ≥ 96%) (Figure 1B, Supplementary Table S1). Increasing x led to decreasing RC of the D-HL(x) materials. Hence, the increasing Si/Al ratio was accompanied by decreasing RC. The D-HL(x) and 0.28Hf-D-HL(x) materials with the same x, possessed similar RC; *e.g.*, 73 and 71% RC for D-HL(0.15) and 0.28Hf-D-HL(0.15), respectively; 22 and 20% RC for D-HL(0.28) and 0.28Hf-D-HL (0.28), respectively. For the yHf-D-HL(0.15) materials, increasing y led to decreasing RC (Supplementary Table S1), which may be partly due to the presence of Hf species inside the pores, causing decreased scattering contrast and reduced intensities of the PXRD peaks. The range of RC of the yHf-D-HL(x) materials with x ≤ 0.15 and y ≤ 0.84 was comparable to that reported by Al-Ani *et al.* (Al-Ani et al., 2019) for hierarchical LTL materials prepared via a surfactant templated approach, at 80–100°C (59–74% RC). The materials yHf-D-HL(0.28) possessed low RC (less than 24%) (Supplementary Table S1).

**FIGURE 3:**
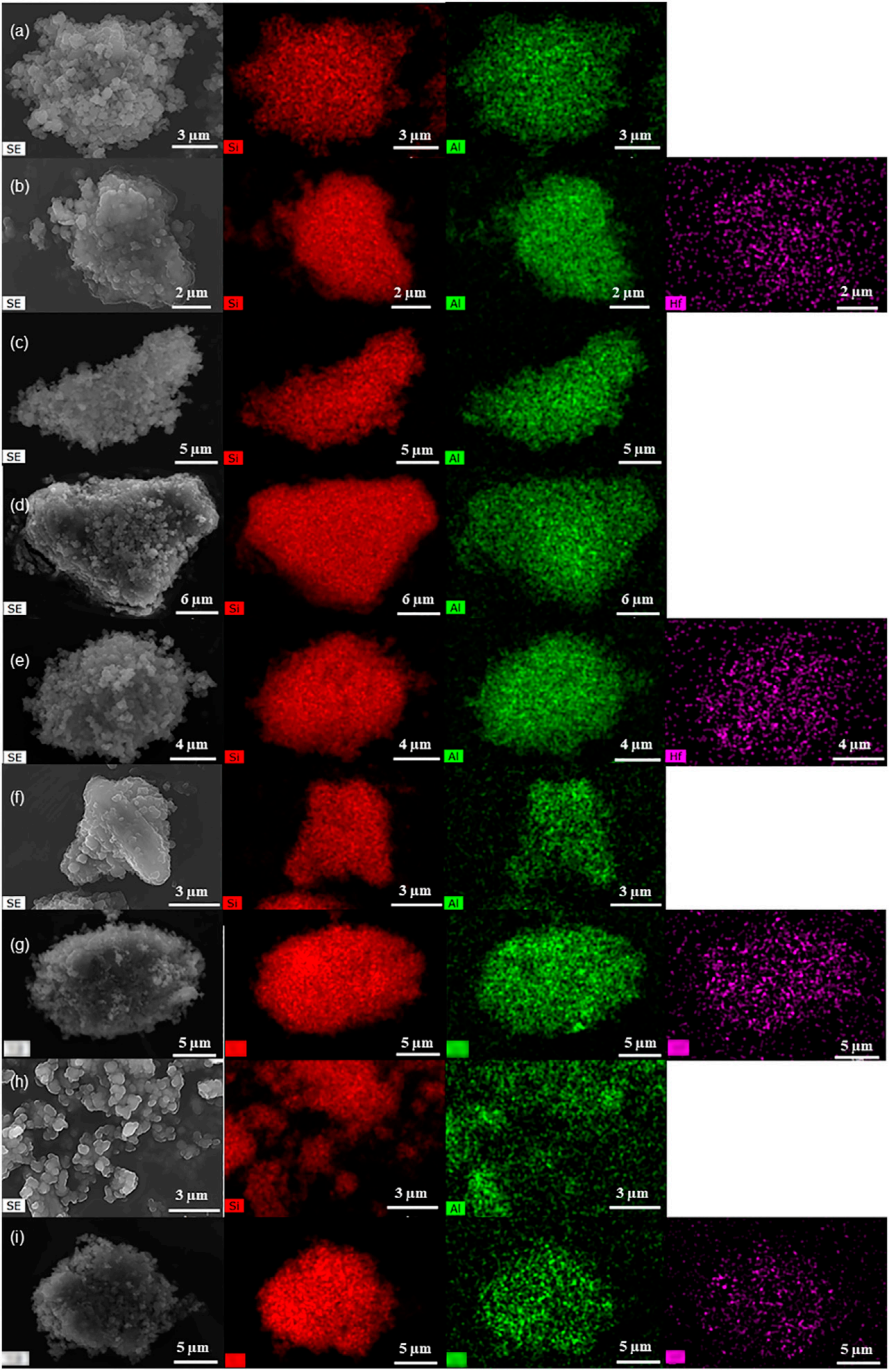
SEM images (left) and element mappings (Si–red, Al–green, Hf–purple) of KL (a), 5Hf-HL (b), D-KL (c), D-HL(x) (x = 0.15 (d), 0.20 (f), 0.28 (h)) and 0.28Hf-D-HL(x) (x = 0.15 (e), 0.20 (g), 0.28 (i)).

The SEM images showed that, in general, the materials consisted of aggregates of small pseudo-spherical particles with sizes of up to *ca.* 30 nm (exemplified for some materials in Figure 3, Supplementary Figure S4, S5). The respective elemental mappings suggested somewhat uniform metal distributions (Figure 3, Supplementary Figure S4, S5) (noteworthy, it was not possible to discriminate HfO_x_ nanocrystallites by SEM).

**FIGURE 4:**
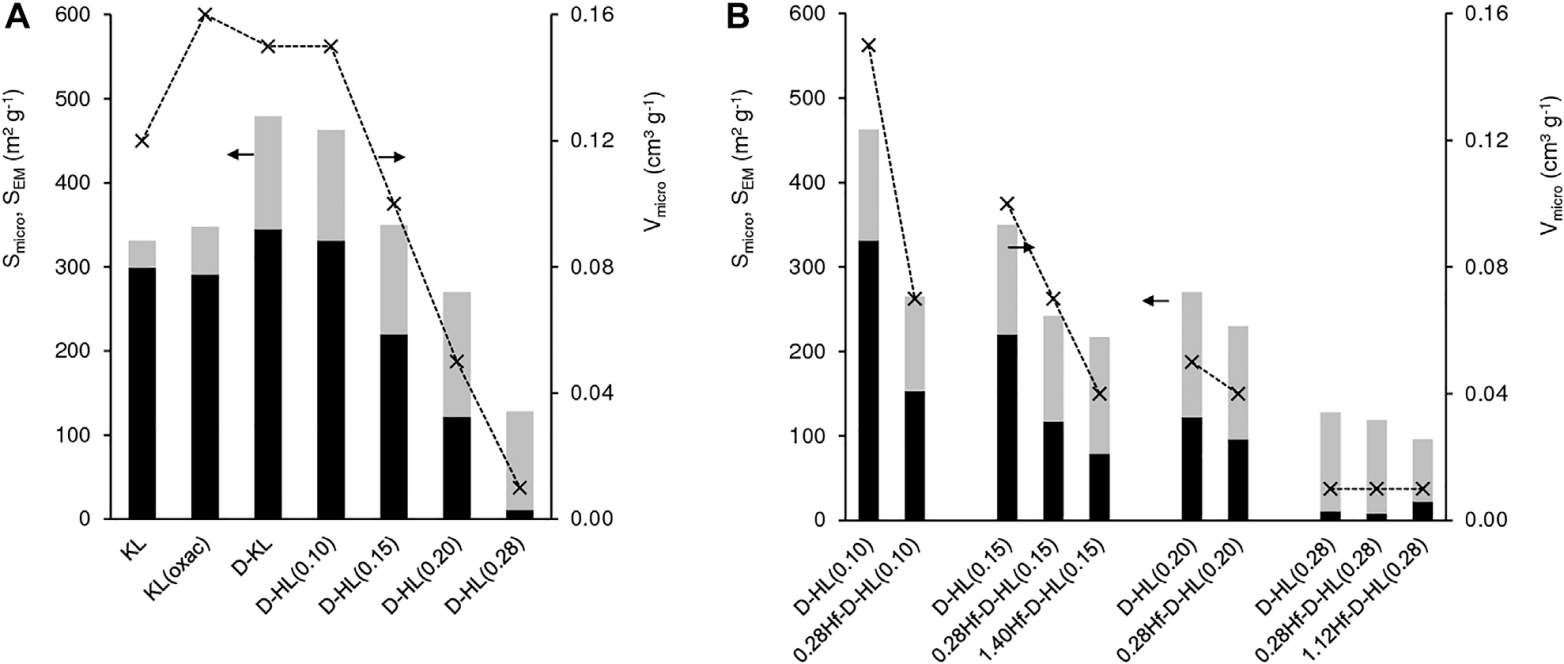
Textural properties of **(A)** KL, KL(oxal), D-KL and D-HL(x), and of **(B)** D-HL(x) and yHf-D-HL(x) materials prepared: micropore specific surface area (S_micro_ (black bars)), external/mesopore specific surface area (S_EM_ (grey bars)) and micropore volume (V_micro_ (*)). The values are given in Table S2.

Commercial KL, HL, and KL(oxac) exhibited type I nitrogen sorption isotherms (Supplementary Figure S6A), characteristic of microporous materials (Gregg and Sing, 1982). The increasing N_2_ uptake as the relative pressure (p/p_0_) approached unity may be attributed to N_2_ sorption on the external surface of the relatively small crystallites. The desilicated materials D-KL and D-HL(x) exhibited type IV isotherms with an inflection point at p/p_0_ > 0.9 (inset of Supplementary Figure S6B), which is characteristic of mesoporous materials (Gregg and Sing, 1982). Whereas commercial KL possessed S_EM_ = 32 m^2^ g^−1^ and V_micro_ = 0.12 cm^3^ g^−1^, the desilicated D-KL material possessed S_EM_ = 134 m^2^ g^−1^ and V_micro_ = 0.15 cm^3^ g^−1^, *i.e.*, the alkaline treatment introduced mesoporosity without affecting significantly V_micro_ (Figure 4A, Supplementary Table S2). Consistently, D-HL(x) exhibited bimodal pore size distributions, in which the mesopore sizes were in the range 2.3–6 nm (Supplementary Figure S6C).

For D-HL(x), increasing x up to 0.20 essentially led to decreasing S_micro_ and V_micro_ without affecting considerably S_EM_ (130–148 m^2^ g^−1^) (Figure 4A, Supplementary Table S2). Materials D-HL(x) with x ≤ 0.15 possessed comparable V_micro_ to literature data for surfactant-templated LTL type materials (Al-Ani et al., 2019). For x = 0.28, V_micro_ and S_micro_ decreased drastically to 0.01 cm^3^ g^−1^ and 11 m^2^ g^−1^, respectively, and the material was essentially mesoporous (S_EM_ = 117 m^2^ g^−1^). Based on these results and PXRD, harsh dealumination seems to cause partial destruction and/or blockage of the micropores (*e.g.*, possibly by inorganic debris formed during post-synthesis treatments).

The introduction of Hf in D-HL(x) with x ≤ 0.20, led to a decrease in S_micro_ and V_micro_, suggesting that Hf sites are at least partly located on the internal micropore surface (Figure 4B, Supplementary Table S2). For x = 0.28, the introduction of Hf led to a decrease in S_EM_ (117 and 74 m^2^ g^−1^ for D-HL(0.28) and 1.12Hf-D-HL(0.28), respectively), suggesting that Hf sites may be located on the mesopore surface.

Furthermore, the micropore size distribution curves of KL showed medians at *ca.* 0.55 and at 0.75 nm, assignable to the 8-MR (3D) and 12-MR (1D) channels. Especially the peak at *ca.* 0.55 nm was significantly attenuated for D-HL(x) with x ≤ 0.20 and negligible for D-HL(0.28) (Supplementary Figure S7A). This was more pronounced after introducing Hf, suggesting that a fraction of Hf species may be located inside micropores and/or at micropore mouths of yHf-D-HL(x) with x ≤ 0.20 (exemplified for 0.28Hf-D-HL(x) in Supplementary Figure S7B).

The ^27^Al MAS NMR spectra of KL, HL, Hf-HL, KL(oxac), D-KL, D-HL(x) and yHf-D-HL(x) with x ≤ 0.15 showed a prominent peak centered at *ca.* 63 ppm, assigned to Al sites in tetrahedral coordination (Al_tetra_) (Supplementary Figure S8). Increasing x above 0.15 led to the broadening of the Al_tetra_ peak and the appearance of a shoulder at *ca.* 53 ppm; the relative intensity of the latter peak increased with increasing x. These results suggest that the decreased crystallinity was accompanied by the formation of broader distributions of Al_tetra_ species (*e.g.*, possessing different bond angles/lengths, coordination spheres). The materials D-HL(x) exhibited a weak resonance at *ca*. 0 ppm due to hexacoordinated (octahedral) Al species (Al_octa_), which was not verified for D-KL, suggesting that Al_octa_ was formed during the acid treatment. A comparison of the materials before and after the introduction of hafnium indicated no significant differences in the Al_tetra_ spectral region, but the Al_octa_ peak disappeared. Hence, it seems that Al_octa_ species were partially linked to the framework of D-HL(x) and converted to Al_tetra_ during SSI/calcination. According to the literature for zeolites, Al_octa_ and Al_tetra_ sites may interconvert (Mafra et al., 2012; Ravi et al., 2021); *e.g.*, hydroxylated-hydrated Al_octa_ sites and hydroxylated Al_tetra_ sites may interconvert (Yakimov et al., 2022). Possibly the two resonances at *ca.* 63 and 53 ppm may include four coordinated hydroxylated (Al(OSi)_4-x_(OH)_x_) and non-hydroxylated (Al(OSi)_4_) aluminum sites.

**FIGURE 5:**
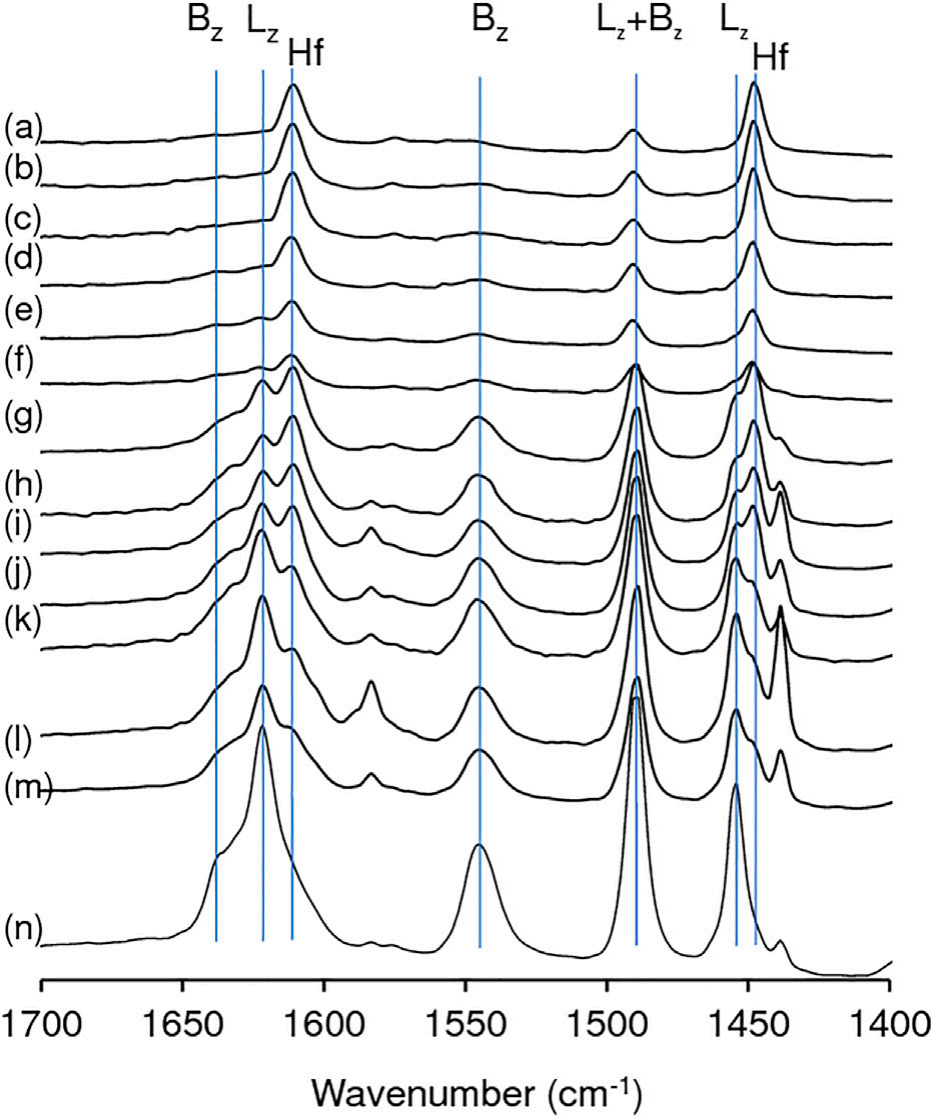
FT-IR spectra of adsorbed pyridine, at 150°C, for yHf-D-HL(0.28) with y = 1.40 (a), 1.12 (b), 0.84 (c), 0.56 (d), 0.28 (e); 0.28Hf-D-HL(0.20) (f); yHf-D-HL(0.15) with y = 1.40 (g), 1.12 (h), 0.84 (i), 0.56 (j), 0.28 (k); 0.28Hf-D-HL(0.10) (l); 0.28Hf-HL (m); HL (n). Hf signalizes the new bands which appeared after Hf introduction; B_z_ and L_z_ signalize typical bands associated with Brønsted and Lewis acid sites of a zeolitic type aluminosilicate framework.

The FT-IR spectrum of adsorbed pyridine for zeolite HL showed the typical bands associated with Al sites possessing Lewis (L) acidity (metal-pyridine complexes, which may possess extra framework metal sites) and Brønsted (B) acidity (pyridinium ions): *ca.* 1,620 cm^−1^ and 1,454 cm^−1^ (L); *ca.* 1,635 cm^−1^ and 1,540 cm^−1^ (B); and 1,490 cm^−1^ (L + B) (Figure 5) (Zhu et al., 2016; Tang et al., 2019; Ravi et al., 2021). A band at 1,439 cm^−1^ may be due to pyridine molecules interacting with weak acid sites. The relative intensities of the bands at 1,454 cm^−1^ (L), 1,540 cm^−1^ (B) and 1,635 cm^−1^ (B) decreased after acid treatment and were hardly distinguishable in the spectra of the materials with x = 0.28. The introduction of hafnium led to the appearance of new bands at *ca.* 1,608 and 1,448 cm^−1^ which became stronger with increasing y up to *ca.* 0.84 mmol_Hf_ g^−1^ (above this value the spectral differences in relative intensities were not so evident). Hence, these two new bands may be attributed to Hf-containing acid sites. The appearance of these new bands after introducing Hf is in agreement with that reported by Tang *et al.* for Hf-USY (Tang et al., 2019). For the yHf-D-HL(0.28) materials, the 1,608 and 1,448 cm^−1^ bands predominated, suggesting that the acidity of these materials was essentially associated with Hf sites.

**FIGURE 6:**
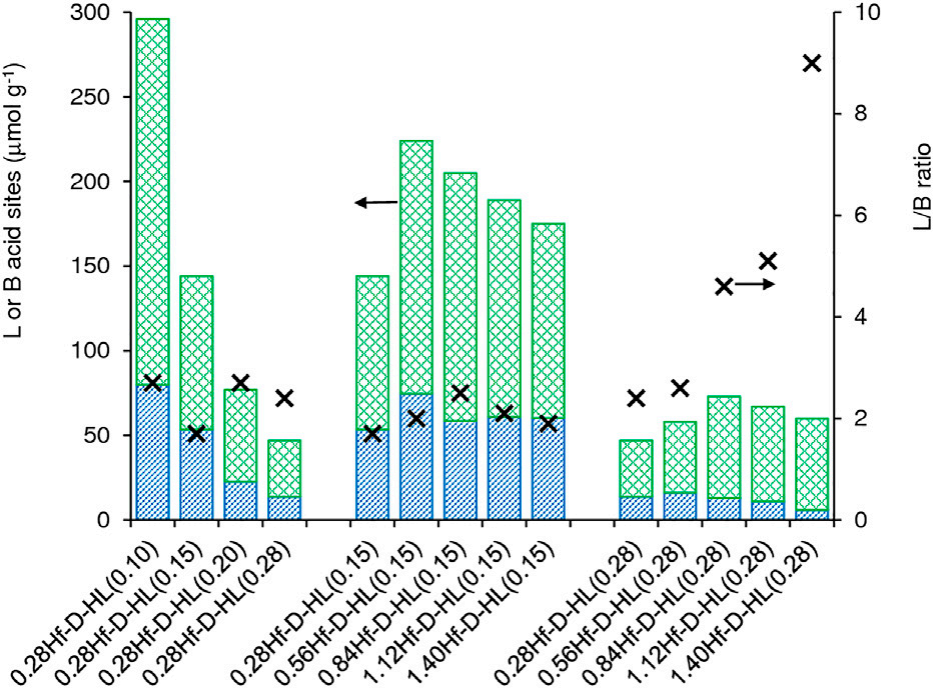
Acid properties of the materials prepared from commercial KL via post-synthesis modifications: L = Lewis acid sites (top green bar), B = Brønsted acid sites (bottom blue bar) and L/B ratio (×). The values are given in Table S3.

Increasing x of the materials 0.28Hf-D-HL(x) led to decreasing amount of L and B acid sites (Figure 6, Supplementary Table S3). On the other hand, the introduction of Hf in HL gave 0.28Hf-HL possessing higher amounts of B and L acid sites (L increased from 82 to 128 μmol g^−1^, and B increased from 45 to 71 μmol g^−1^, respectively (Supplementary Table S3)). These results parallel those reported in the literature for Beta zeolites with and without hafnium (Antunes et al., 2022a).

For each set of materials yHf-D-HL(0.15) and yHf-D-HL(0.28), a maximum amount of L acid sites was reached as a function of y; specifically, at y = 0.56 and 0.84 for materials with x = 0.15 and 0.28, respectively (Figure 6, Supplementary Table S3). For the lower range of Hf loading (y), the increasing L acidity with increasing y suggests that uniformly distributed Hf-sites were introduced. On the other hand, for the higher range of Hf loadings, L may decrease due to 1) formation of dimeric/oligomeric Hf species which may possess reduced acidity and/or hinder the access of pyridine molecules to some acid sites of the aluminosilicate support, and 2) hafnium oxide nanoparticles formed at higher y may possess inaccessible Hf sites. For yHf-D-HL(0.15), the L/B ratios (1.7–2.5) did not vary considerably with y, whereas for yHf-D-HL(0.28) the L/B ratio (2.4–9.0) increased considerably with increasing y in the range 0.28–1.68 mmol_Hf_ g^−1^ (Figure 6).

In general, the yHf-D-HL(x) materials possessed moderate to strong L acid sites (L_350_/L_150_ up to *ca.* 0.33) and did not possess strong B acid sites (B_350_/B_150_ ≅ 0), Supplementary Table S3. For the 0.28Hf-D-HL(x) materials (*i.e.*, Si/Al varied and Hf load was similar), the L acid strength increased with increasing x above 0.15 M H_2_SO_4_ (L_350_/L_150_ was similar for x = 0.10 and 0.15). On the other hand, the influence of y on the L acid strength was not straightforward: for yHf-D-HL(0.15), the L acid strength decreased with increasing y, whereas for yHf-D-HL(0.28) no straightforward relationship could be established. The acid properties may depend on several factors. The PXRD data indicated that condensed hafnium oxide species may be formed at higher y, which may impact on the amount and strength of accessible acid sites. The materials yHf-D-HL(x) may possess different metal (Al, Hf) acid sites, chemical structures and coordination spheres, bond angles/lengths and binding energies (*e.g.*, distorted geometries), *etc*. For example, according to the literature, framework Lewis acid Hf sites may be hydrolyzed or non-hydrolyzed species, and the former may be stronger than the latter (Tang et al., 2019).

### 3.2 Catalytic studies

As mentioned in the Introduction, HMF, AnL and LA may be formed in carbohydrate conversion processes, and converted to GVL under acid and reduction conditions, and thus it is interesting to study their reactivities, envisaging future integrated reaction systems over multifunctional catalysts. The acid sites of the aluminosilicate LTL framework may promote the acid reactions (*e.g.*, esterification, etherification). On the other hand, desilication and partial dealumination of the framework may form vacant sites for introducing hafnium via SSI, furnishing the zeolite with CTH activity. In this fashion, multifunctional LTL type catalysts may be produced, capable of promoting several paths that lead to target bioproducts.

To study the influence of the post-synthesis conditions, the catalytic studies consisted of firstly studying the influence of the acid treatment (dealumination degree) of D-HL(x) (x = 0.10, 0.15, 0.20 or 0.28 M H_2_SO_4_) on the LA reaction, keeping constant the amount of impregnated hafnium (y = 0.28 mmol_Hf_ g^−1^). Subsequently, the influence of the Hf loading (y in the range 0.28–1.68 mmol_Hf_ g^−1^) was studied for two sets of materials, namely (crystalline micro/mesoporous) yHf-D-HL(0.15) and (mostly amorphous, mesoporous) yHf-D-HL(0.28). Kinetic, mechanistic, catalyst stability and finally substrate scope (AnL, HMF) were studied for selected catalysts.

#### 3.2.1 LA to GVL

##### 3.2.1.1 General considerations

**FIGURE 7:**
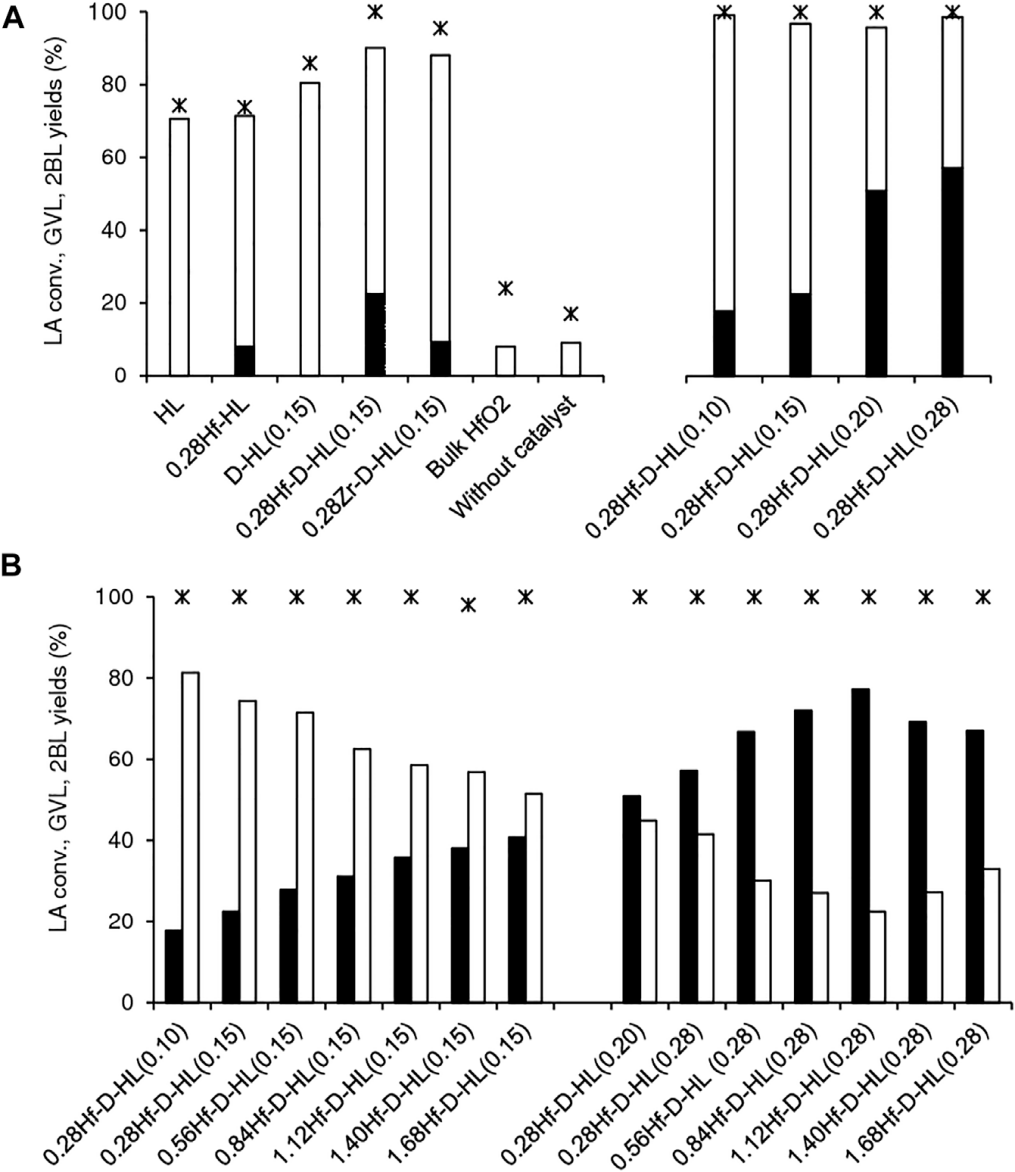
Catalytic performances of the prepared catalysts **(A,B)** for LA conversion (*) to GVL (black bars) and 2BL (white bars). Tests without catalyst or in the presence of HfO_2_ are included for comparison **(A)**. Reaction conditions: 0.45 M LA in 2BuOH, 200°C, 24 h.

The reaction of LA in the presence of the yHf-D-HL(x) materials gave GVL and 2BL in a high total yield (>90%) with molar ratios GVL/2BL in the range 0.2–3.5, at 200°C, 24 h. Without a catalyst, the reaction was much slower and GVL was not formed, under similar conditions (17% conversion, 9% 2BL yield) (Figure 7).

Literature studies suggest that the conversion of LA to GVL in alcohol medium may be favoured by L and B acidity (Li et al., 2017; Xu et al., 2017; Li et al., 2018; Tang et al., 2019; He et al., 2020; Li et al., 2021), such as Lewis acid Hf sites (Kumar et al., 2016; Tang et al., 2019). A comparative study for the aluminosilicates HL and D-HL(0.15) (both prepared via acid treatment using x = 0.15 M H_2_SO_4_), and the respective Hf-containing materials 0.28Hf-HL and 0.28Hf-D-HL(0.15), indicated that the aluminosilicates effectively converted LA to 2BL, but failed to give GVL (Figure 7A). The formation of GVL required the presence of Hf sites. Nevertheless, bulk HfO_2_ led to similar results to the blank test without catalyst (24% LA conversion, 8% 2BL yield, and GVL was not formed), suggesting that the type of Hf sites may be determinant for GVL formation.

The zirconium catalyst 0.28Zr-D-HL(0.15) was less effective than its counterpart 0.28Hf-D-HL(0.15); the former led to less than half the GVL yield reached in the presence of 0.28Hf-D-HL(0.15) (9 and 22% GVL yield, at 200°C/24 h) (Figure 7A). Hence, hafnium catalysts seem more promising for the target reaction, which parallels literature studies for Hf- versus Zr-Beta (Antunes et al., 2022a), and Hf- versus Zr- and Sn-USY (Tang et al., 2019).

A comparative study for (non-desilicated) 0.28Hf-HL and (desilicated) 0.28Hf-D-HL(0.15) (which possessed the same Hf loading (y = 0.28 mmol_Hf_ g^−1^) and were subjected to similar acid treatment using x = 0.15 M H_2_SO_4_) indicated that the desilicated catalyst 0.28Hf-D-HL(0.15) was more active than 0.28Hf-HL (100 74% and LA conversion, respectively), led to higher 2BL + GVL total yield (90 and 71%, respectively) and higher GVL/2BL molar ratio (0.33 and 0.13, respectively) (Figure 7A), suggesting a favourable effect of desilication on the catalytic performance for targeting GVL.

##### 3.2.1.2 Influence of acid treatment (x) and Hf loading (y)

**FIGURE 8:**
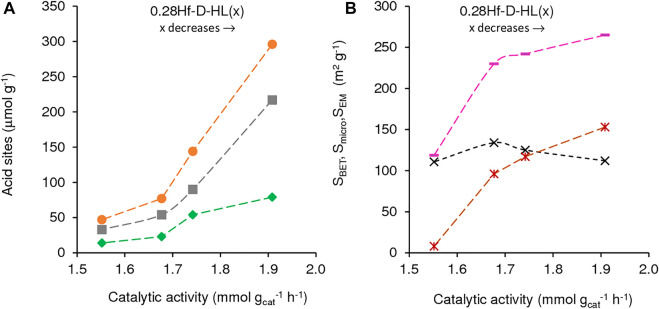
Influence of **(A)** the amount of acid sites (L (■), B (♦) and L + B (•)) and **(B)** textural properties (S_BET_ (-), S_EM_ (×), S_micro_ (*) on catalytic activity of 0.28Hf-D-HL(x) for LA conversion. Reaction conditions: 0.45 M LA in 2BuOH, 25.5 g_cat_ L^−1^, 200°C, 5 h.

**FIGURE 9:**
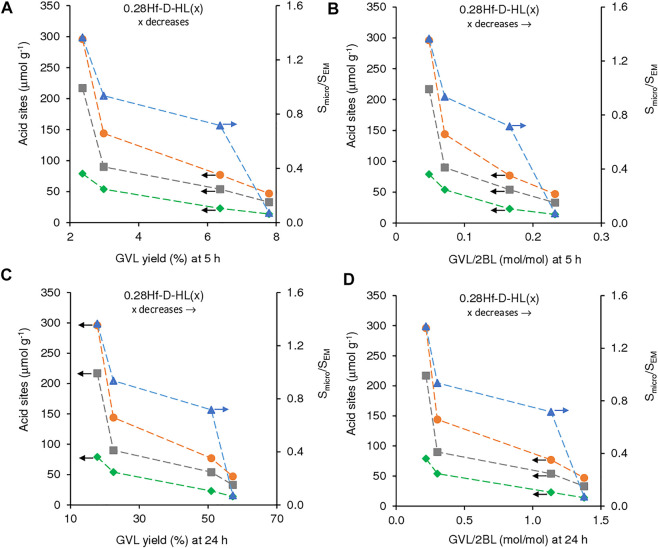
Influence of the amount of acid sites (L (■), B (♦) and L + B (•)) and S_micro_/S_EM_ ratio (▲), on GVL yield (**(A)**, **(C)**) and GVL/2BL molar ratio (**(B)**, **(D)**), at 5 h (**(A)**, **(B)**) and 24 h (**(C)**, **(D)**) for the reaction of LA in the presence of 0.28Hf-D-HL(x). Reaction conditions: 0.45 M LA in 2BuOH, 25.5 g_cat_ L^−1^, 200°C.

The influence of the acid treatment (dealumination degree) on the catalytic performance was studied keeping constant the Hf loading (y = 0.28 mmol_Hf_ g^−1^), *i.e.*, comparing the 0.28Hf-D-HL(x) catalysts (Figure 8, Figure 9). Catalysts 0.28Hf-D-HL(x) led mainly to 2BL and GVL, which were formed in high total yield (96–99%, Figure 7), with GVL/2BL molar ratios in the range 0.2–1.4, at 24 h, 200°C. The activity (mmol g_cat_^−1^ h^−1^, based on conversion at 5 h) increased with decreasing x in the order 1.55 (x = 0.28) < 1.68 (x = 0.20) < 1.74 (x = 0.15) < 1.91 (x = 0.10), which correlated with the increasing amounts of B and L acid sites (Figure 8A) and increasing S_BET_ and S_micro_ (Figure 8B); S_EM_ was roughly constant, and no clear correlation could be established with the L/B ratio.

Although the activity of 0.28Hf-D-HL(x) decreased with increasing x, GVL yield and GVL/2BL ratios at 5 and 24 h increased (Figure 9). This may be partly due to a levelling off effect of the lower amounts of total acid sites for higher x, by a lower ratio S_micro_/S_EM_ which may avoid steric hindrance effects, favouring GVL formation.

Further studies were carried out to check the influence of the Hf loading (y), specifically for the two sets of materials yHf-D-HL(x) with x = 0.15 and 0.28. yHf-D-HL(0.15) led mainly to 2BL (51–71% yield), and GVL was formed in 22–38% yield at 200°C/24 h (Figure 7B), whereas yHf-D-HL(0.28) led mainly to GVL (57–77% yield), and 2BL was formed in 22–45% yield at 200°C/24 h (Figure 7B).

**FIGURE 10:**
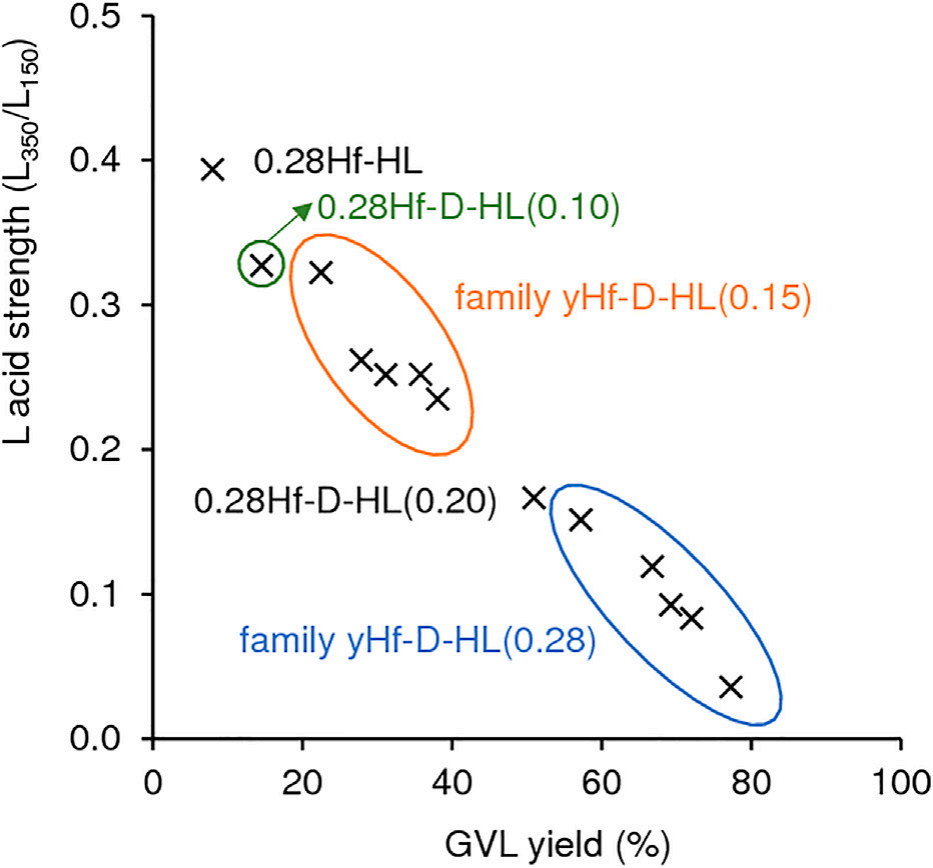
Influence of the L acid strength (based on the ratio L_350_/L_150_) on GVL yields for the Hf-containing catalysts. Reaction conditions: 0.45 M LA in 2BuOH, 25.5 g_cat_ L^−1^, 200°C, 24 h.

According to the literature, stronger L acidity may enhance CTH activity (Li et al., 2017). However, taking into consideration all the Hf-containing materials prepared (yHf-D-HL(x), 0.28Hf-HL), GVL yield increased somewhat linearly with decreasing L acid strength (*R* ^2^ = 0.9835) (Figure 10). These results may be due to an interplay of several factors. For example, for materials with y = 0.28 mmol_Hf_ g^−1^, the L acid strength increased with increasing S_micro_/S_EM_ (Supplementary Table S2, S3); specifically, L_350_/L_150_ was 0.39 (0.28Hf-HL) > 0.33 (0.28Hf-D-HL(0.10)) > 0.32 (0.28Hf-D-HL(0.15)) > 0.17 (0.28Hf-D-HL(0.20)) > 0.15 (0.28Hf-D-HL(0.28)), and S_micro_/S_EM_ followed a similar order, 2.21 (0.28Hf-HL) > 1.37 (0.28Hf-D-HL(0.10)) > 0.94 (0.28Hf-D-HL(0.15)) > 0.72 (0.28Hf-D-HL(0.20)) > 0.07 (0.28Hf-D-HL(0.28)). Hence, for these materials with y = 0.28 mmol_Hf_ g^−1^, lower S_micro_/S_EM_ may be a main factor in favour of GVL formation. For the remaining materials with y > 0.28 mmol_Hf_ g^−1^, the L acid strength dependency on y (and thus on GVL yields) was not straightforward; higher y may lead to relevant differences in surface chemistry, impacting on catalytic performances.

##### 3.2.1.3 Mechanistic studies

In the conversion of LA to GVL, the secondary alcohol (2BuOH) plays multiple roles, *i.e*., as solvent and as reducing, etherification and esterification agent. The acid-catalyzed esterification of LA gives alkyl levulinates (LE), and the CTH of LA and LE may give 4-hydroxypentanoic acid (HPA) and alkyl 4-hydroxypentanoates (HPE), respectively, *i.e.*, the carbonyl group in position C4 is reduced to an alcohol group. Literature studies reported that HPA/HPE may undergo etherification at the alcohol group in position C4, giving 4-alkoxypentanoic acid (APA) and alkyl 4-alkoxypentanoates (APE), respectively (Tang et al., 2015; Li et al., 2016a; Valekar et al., 2016; Wang et al., 2017). Although HPA/HPE/APA/APE were not identified for the yHf-H-DL(x) catalysts, one cannot exclude their possible formation and subsequent lactonization (and dealkoxylation in the case of HPE/APE) to give GVL (Li et al., 2016a; Wang et al., 2017). According to the literature, even the intrinsic acidity of LA may promote relatively fast lactonization of HPA (Xie et al., 2016; Winoto et al., 2019).

To gain mechanistic insights, the kinetic curves of the reaction of LA were measured for (crystalline, micro/mesoporous) 1.40Hf-D-HL(0.15) and (mostly amorphous, mesoporous) 1.12Hf-D-HL(0.28), at 200°C (Supplementary Figure S9). For the two catalysts, the LA conversion versus time profiles were roughly coincident, and it was verified an approximately linear dependency (*R* ^*2*^ ≥ 0.995) of ln ([LA]_0_/[LA]) on reaction time (considering the integrated rate law: ln ([LA]_0_/[LA]) = *k* t, where *k* is the kinetic constant and *t* is reaction time) (Supplementary Figure S9A). These results suggest that the reaction rate was apparently first order in LA concentration. Luo *et al.* reported a first order dependency on substrate concentration, for the reaction of LE to GVL over Hf-zeolites in 2BuOH (Luo et al., 2014).

**FIGURE 11:**
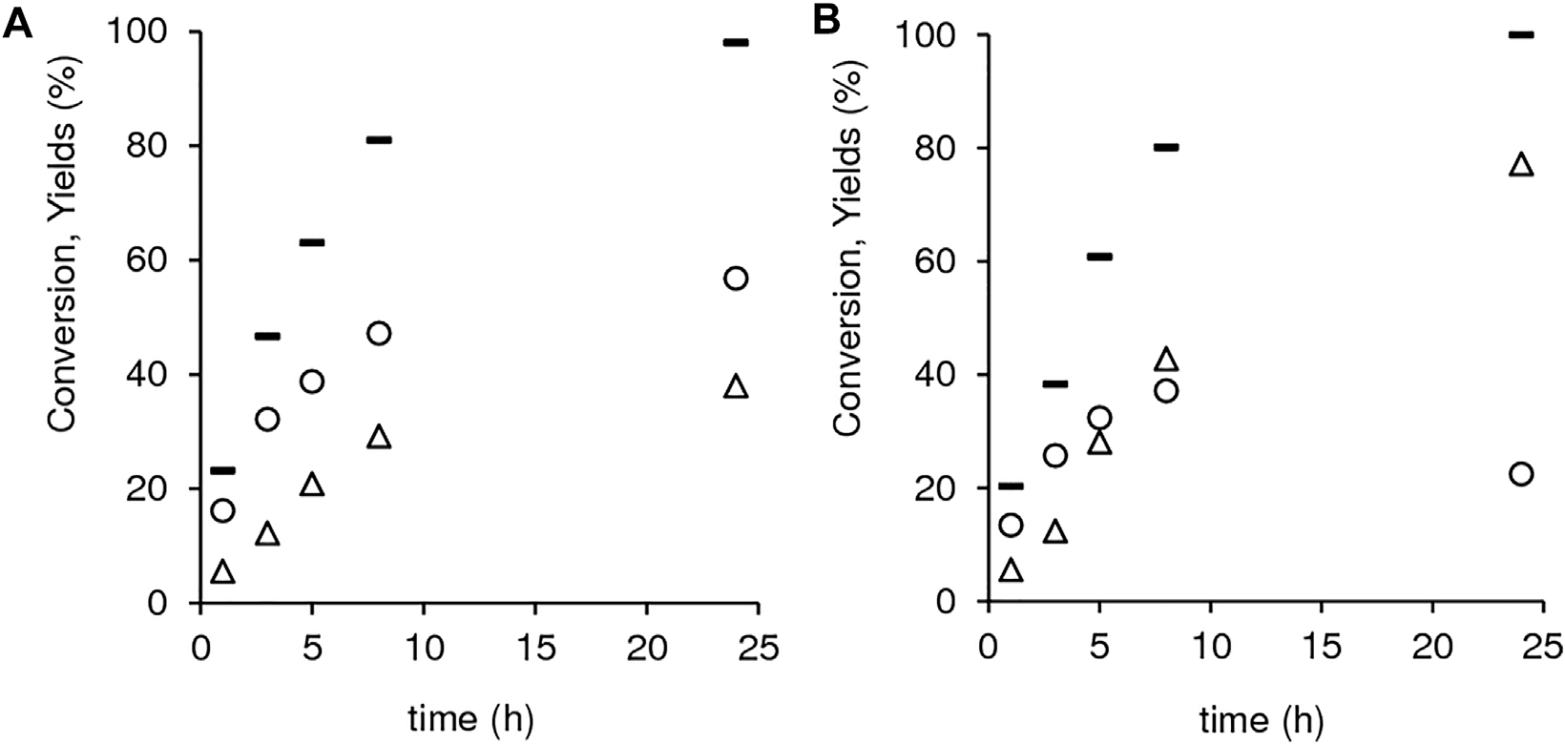
Dependency of LA conversion (-), GVL yield (Δ) and 2BL yield (o) on reaction time for 1.40Hf-D-HL(0.15) **(A)** and 1.12Hf-D-HL(0.28) **(B)**. Reaction conditions: 0.45 M LA in 2BuOH, 25.5 g_cat_ L^−1^, 200°C.

Despite the similar catalytic activities, the two materials exhibited different kinetic curves of GVL and 2BL formation (Figure 11). For 1.40Hf-D-HL(0.15), GVL and 2BL were formed in parallel until *ca.* 98% conversion (Figure 11A), suggesting that GVL may be formed from LA without the intermediate formation of 2BL. Somewhat parallel formation of GVL and 2BL was also verified for 1.12Hf-D-HL(0.28) in an initial stage, but as LA conversion increased from 80 to 100% (20% difference), the 2BL yield dropped from 37 to 22% (15% difference) and GVL yield increased considerably from 43 to 77% (34% difference) (Figure 11B). The increment in GVL yield (34%) was approximately equal to the total consumption of LA plus 2BL (35%) in the same time interval (8–24 h), suggesting that both LA and 2BL were converted to GVL.

One cannot rule out the hypothesis of other intermediates, besides 2BL, being involved in the formation of GVL. For example, the reaction of LA in the presence of 1.12Hf-D-HL(0.28) using lower [LA]_0_ of 0.11 M, was complete within 1 h and, yet GVL continued to be formed (64–91% GVL yield between 1 and 5 h), while 2BL concentration remained approximately constant (9–10% 2BL yield between 1 and 5 h) (Supplementary Figure S9B). Hence, it seems that the intermediates to GVL were not fully quantified. Possible explanations for this include: 1) some intermediates may be essentially adsorbed on the catalyst´s surface and thus not analyzed in the liquid bulk; 2) some intermediates may be unstable under the analytical conditions.

In summary, 1.12Hf-D-HL(0.28) seems to effectively promote GVL formation via intermediate formation of 2BL. This catalyst possessed lower S_micro_/S_EM_ than 1.40Hf-D-HL(0.15) (*ca.* 0.3 and 0.6, respectively), which may facilitate reaction pathways involving bulkier intermediates and transition states, *e.g.*, LE/HPE/APE are bulkier than LA/HPA/APA, respectively. Luo *et al.* reported for methyl levulinate conversion to GVL over Hf-Beta, in 2BuOH, a dual-binding mechanism where the alkyl levulinate and the alcohol H-donor interacted (involving a hydride shift) with a single metal site forming a voluminous six-membered transition state (Luo et al., 2014). Accordingly, steric hindrance may become important for catalysts possessing higher S_micro_/S_EM_.

##### 3.2.1.4 Comparisons between yHf-D-HL(x) and other materials

Based on the above results, it is interesting to compare the catalytic performances of yHf-D-HL(x) to hafnium silicates possessing high mesopore surface area, such as the mesoporous hafnium silicate Hf-TUD-1(50), previously described by our group (Antunes et al., 2021). Hf-TUD-1(50) possessed a similar Hf load of 0.31 mmol_Hf_ g^−1^ to 0.28Hf-D-HL(0.28), but higher L + B = 130 μmol g^−1^, L/B = 12 and mesopore specific surface area of *ca.* 660 m^2^ g^−1^ (Antunes et al., 2021); for 0.28Hf-D-HL(0.28), L + B = 47 μmol g^−1^, L/B = 2.4 and S_BET_ = 119 m^2^ g^−1^. Hf-TUD-1(50) led to 29% GVL yield at 200°C/24 h (Supplementary Table S4, entry 6), which was approximately half of that for 0.28Hf-D-HL(0.28) (57% yield), under similar reaction conditions. Hence, the enhanced mesopore surface area of Hf-TUD-1(50) was not sufficient to warrant high GVL yield. Catalyst 0.28Hf-D-HL(0.28) possesses distinct surface chemistry partly because it has in its genesis an LTL type (starting) material.

To the best of our knowledge, this is the first study for LTL zeotype catalysts for LA conversion to GVL. Supplementary Table S4 further compares the catalytic performance of yHf-D-HL(x) to literature data for the reaction of LA to GVL, in the presence of micro/mesoporous zeotypes or mesoporous silicas/silicates, using an alcohol as reducing agent (Kuwahara et al., 2014; Antunes et al., 2015, 2016a, 2016b; Hengne et al., 2016; Winoto et al., 2016; Kuwahara et al., 2017; Xu et al., 2017; Zhou et al., 2018; Kumar and Srivastava, 2019; Morales et al., 2019; Tang et al., 2019; He et al., 2020; Kumaravel et al., 2020; López-Aguado et al., 2020; García et al., 2021; Ostovar et al., 2021; Antunes et al., 2022a). The different studies were carried out using different reaction conditions, making it difficult to establish clear comparisons. Without considering the differences in reaction conditions, and based solely on GVL yields, the best result for 0.28Hf-D-HL(0.28) (91% GVL yield, entry 5) was comparable to some of the best results indicated in Supplementary Table S4. GVL yields of at least 90% were reported for micro/mesoporous zeotypes such as Hf-WdeSAlBeta-m (entry 7) (Antunes et al., 2022a), Hf-USY (entry 17) (Tang et al., 2019) and Zr-AlBeta (entry 11) (López-Aguado et al., 2020) which led to 99% (180°C/24 h), 95% (150°C/10 h) and 92% GVL yield (170°C/6 h), respectively, and some Zr-containing mesoporous SBA-15 type catalysts (synthesized using the relatively expensive polymeric template Pluronic P123) which led to 90–95% GVL yield (entries 19, 20) (Kuwahara et al., 2014, 2017; Zhou et al., 2018). Catalyst 1.12Hf-D-HL(0.28) led to 64% GVL yield at 200°C/1 h (Figure 9B), which was higher than that reported for Ni-Sepiolite (<1% yield at 180°C/2 h, entry 25) (García et al., 2021). Moderate GVL yields were reported for a composite Zr-AlBeta/TUD-1 (31% at 150°C/72 h, entry 13) (Antunes et al., 2016b) and mesoporous Zr (7.6wt%)-SBA-15-SGD (33% at 160°C/6 h, entry 18) (Ostovar et al., 2021). GVL was not formed for Zr-TUD-1 and ZrAl-TUD-1 at 120°C/24 h (entries 14 and 15, respectively) (Antunes et al., 2016b; 2016a).

##### 3.2.1.5 Catalyst stability

The fresh and used catalysts 0.28Hf-D-HL(x) (x = 0.10, 0.15, 0.20, 0.28) exhibited similar (white) colour, and the material balances closed in at least 91 mol%, considering GVL and 2BL as the useful bioproducts. ATR FT-IR spectroscopy indicated the presence of carbonaceous matter in the solids (exemplified for 0.28Hf-D-HL(0.15) in Supplementary Figure S10). The used catalyst exhibited new weak bands which were not verified for the fresh catalyst; *ca.* 1,460 cm^−1^ assignable to C-H vibrations of adsorbed organic matter (O´Dell et al., 2008), *ca.* 1,377 cm^−1^ assignable to metal-alkoxide type groups (Lynch et al., 1964), *ca.* 1,700 cm^−1^ which may be associated with the carbonyl moiety of carboxylic acid or ester functional groups (Nandiyanto et al., 2019), and *ca.* 1,400 cm^−1^ assignable to carboxylate groups (Nandiyanto et al., 2019). The thermal treatment of the catalyst at 550°C led to the disappearance of these new bands, and the spectrum was similar to that of the original catalyst. Hence, the surface chemistry was preserved during the catalytic and thermal regeneration processes. Furthermore, the PXRD patterns of the thermally regenerated solids were similar to those of the respective original catalysts (Supplementary Figure S11).

Elemental analysis of the washed-dried 0.28Hf-D-HL(x) catalysts (after a 5 h batch run) indicated that the carbon content (wt% C) increased with decreasing x, in the order 1.2 wt% (x = 0.28) < 1.7 wt% (x = 0.20) < 2.8 wt% (x = 0.15) < 3.7 w% (x = 0.10). While enhanced adsorption of carbonaceous matter did not seem to negatively affect LA conversion (54, 49, 47 and 44% conversion for x = 0.10, 0.15, 0.20 and 0.28, respectively), it led to decreasing GVL yields and GVL/2BL ratios.

**FIGURE 12:**
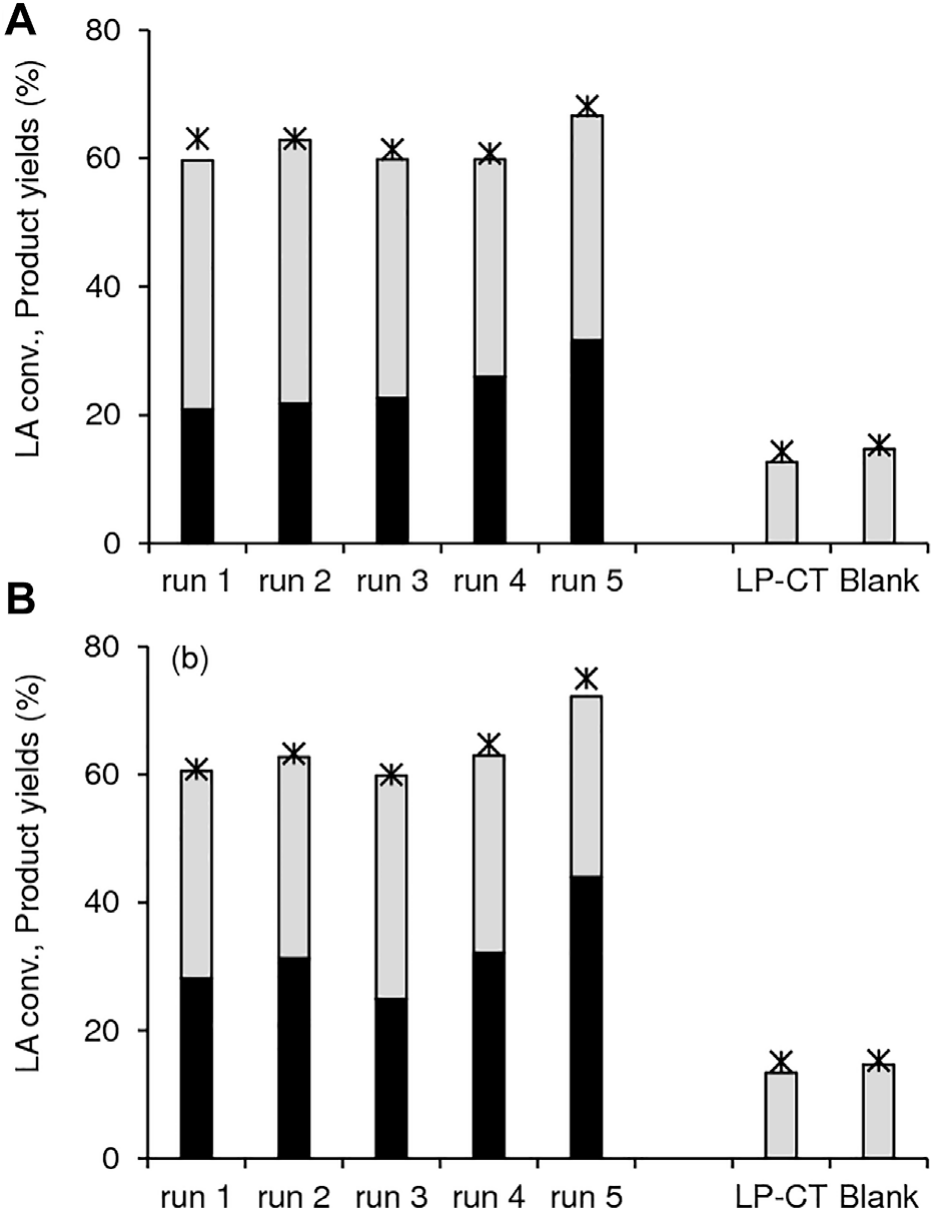
Conversion of LA (*) and yields of GVL (black bars) and 2BL (grey bars) in the presence of **(A)** 1.40Hf-D-HL(0.15) and **(B)** 1.12Hf-D-HL(0.28). The contact tests (LP-CT) for each catalyst as well as the blank test (without catalyst at 5 h) are included for comparison. Reaction conditions: 0.45 M LA in 2BuOH, 25.5 g_cat_ L^−1^, 200°C, 5 h.

The thermally regenerated solids 1.40Hf-D-HL(0.15) and 1.12Hf-D-HL(0.28) were reused and performed somewhat steadily for five consecutive 5 h-batch runs at 200 °C (Figure 12). For the two catalysts, the contact tests (details in the experimental section) indicated that LP-CT led to similar results to those without catalyst; 14–15% LA conversion, 13–15% 2BL yield, and GVL was not formed (Figure 12). These results suggest that no active species were leached from the catalysts. Consistently, EDS of the fresh and used catalysts indicated that Si/Al and Si/Hf remained similar: for fresh and used 1.40Hf-D-HL(0.15), Si/Al = 8 and Si/Hf = 8; for fresh and used 1.12Hf-D-HL(0.28), Si/Al = 40 and 44, respectively, and Si/Hf = 9 for the two solids. Not only the PXRD patterns (Supplementary Figure S11), but also the morphology and metal distributions (Supplementary Figure S12), textural and acid properties (Supplementary Table S5) of the fresh and used catalysts were roughly comparable, suggesting that the materials were relatively stable.

#### 3.2.2 Reaction of α-angelica lactone

The reaction of AnL to GVL involves acid and reduction chemistry (*e.g.*, hydration, CTH and lactonization) (Li et al., 2018; Antunes et al., 2022a, 2022b). According to the literature, the reversible reaction between AnL and LA may occur via hydration-dehydration (Dutta and Bhat, 2021; Liu et al., 2021; Yuan et al., 2021), but AnL was not detected in the experiments using LA as substrate, whereas LA was formed using AnL as substrate. This is in agreement with literature studies for Hf-containing catalysts in alcohol media (Al-Shaal et al., 2015; Li et al., 2018; Antunes et al., 2022b, 2022a).

The reaction of AnL in the presence of HL and D-HL(0.15) gave LA and 2BL in high total yields (88 and 97% yield, respectively, at *ca.* 100% conversion, 200 °C/24 h), but no GVL was formed (Table 1). Hence, the aluminosilicate supports were essentially effective for converting AnL to 2BL.

**TABLE 1:** Performance of the prepared catalysts in the reaction of α-angelica lactone.^a^

Catalyst	AnL conversion (%)	Product yields (%)			
		LA	GVL	2BL	Total
HL	99	14	0	74	88
0.28Hf-HL	100	15	9	67	91
0.28Hf-D-HL(0.10)	98	0	18	75	93
D-HL(0.15)	98	8	0	89	97
0.28Hf-D-HL(0.15)	98	0	29	64	93
1.40Hf-D-HL(0.15)	99	0	34	60	94
0.28Hf-D-HL(0.20)	99	0	38	57	95
0.28Hf-D-HL(0.28)	100	0	45	49	94
1.12Hf-D-HL(0.28)	100	0	54	43	97

a Reaction conditions: 0.45 M AnL in 2BuOH, 25.5 g_cat_ L^−1^, 200 °C, 24 h. β-Angelica lactone was formed for HL (0.8% yield); no measurable amounts of this isomer were obtained for the remaining catalytic tests.

The introduction of hafnium was required to confer CTH activity for GVL formation (Table 1). A comparison of 0.28Hf-HL (non-desilicated) and 0.28Hf-D-HL(0.15) (desilicated), both subjected to the same acid treatment (x = 0.15 M H_2_SO_4_), indicates a favourable effect of desilication on GVL yield; 9 and 29% for the non-desilicated and the desilicated materials, respectively, at 91–93% AnL conversion, 200°C/24 h.

Increasing x from 0.10 to 0.28 led to 2BL plus GVL, which were formed in a total yield in the range 93–94% at 98–100% AnL conversion (Table 1). In parallel to that verified with LA as substrate, the GVL/2BL ratio (and GVL yields) increased with decreasing S_micro_/S_EM_ (increasing x) (Supplementary Figure S13A).

To check the influence of the Hf loading on the AnL reaction, comparative studies were carried out for yHf-D-HL(0.15) with y = 0.28 and 1.40, and, on the other hand, for yHf-D-HL(0.28) with y = 0.28 and 1.12 (Table 1). For each pair of materials with equal x, the GVL/2BL ratio and GVL yields were higher for the catalyst with higher y; the best results were obtained for 1.12Hf-D-HL(0.28), *i.e.*, 54% GVL yield and GVL/2BL = 1.26, at 100% AnL conversion, 200 °C/24 h.

#### 3.2.3 Reaction of 5-(Hydroxymethyl)furfural

Catalysts yHf-D-HL(x) were further explored for the reaction of HMF (Table 2). The aluminosilicates HL and D-HL(0.15) led to somewhat comparable results; 5-methylfurfural (5MF), 2BL and 5-(*sec*-butoxymethyl)furfural (BMF) were formed in total yields of 91 and 85%, at 95 and 90% HMF conversion, respectively, 200°C/24 h. The main product was BMF (73–75% yield) which may be formed via etherification of HMF. HMF to BMF may be enhanced by B acid sites (Ly et al., 2017). BMF may undergo acid-catalyzed ring-opening to 2BL, although the latter was formed in only 4–6% yield. On the other hand, 5MF was formed in 8–10% yield. Ly *et al.* reported minor amounts of 5MF formed in the reaction of HMF in the presence of Al/SiO_2_ (aluminium supported on a commercial silica) in 2BuOH at 180°C/6 h (Ly et al., 2017). Elsayed *et al.* reported HMF to 5MF in 1-butanol, at 200°C, in the presence of Zr-Fe magnetic supported on activated carbon (Elsayed et al., 2021). Hence, 5MF may be a by-product in CTH/alcohol systems.

**TABLE 2:** Performance of the prepared catalysts in the reaction of 5-(hydroxymethyl)furfural (HMF).^a^

Catalyst	HMF conversion (%)	Product yields (%)				
		5MF	2BL	BMF	BBMF	Total
HL	95	10	6	75	0	91
0.28Hf-HL	77	14	3	35	8	90
0.28Hf-D-HL(0.10)	92	9	2	59	4	74
D-HL(0.15)	90	8	4	73	0	85
0.28Hf-D-HL(0.15)	85	11	1	54	7	73
1.40Hf-D-HL(0.15)	68	9	1	10	7	16
0.28Hf-D-HL(0.20)	79	12	1	48	12	73
0.28Hf-D-HL(0.28)	71	22	4	20	21	67
1.12Hf-D-HL(0.28)	60	12	2	13	12	39

a Reaction conditions: 0.45 M HMF, in 2BuOH, 25.5 g_cat_ L^−1^, 200°C, 24 h.

The introduction of hafnium in the materials led mainly to BMF (*e.g.*, 35 and 54% yield for (non-desilicated) 0.28Hf-HL and (desilicated) 0.28Hf-D-HL(0.15), respectively). 2BL was formed in very low amounts, and thus the subsequent conversion of 2BL to GVL did not take place to a measurable extent (Table 2). The diether 2,5-bis(*sec*-butoxymethyl)furan (BBMF) was formed in up to 21% yield at 200°C/24 h, only in the presence of the Hf-containing catalysts. This path may involve integrated acid (etherification) and CTH (carbonyl group reduction) reactions.

Increasing x for the 0.28Hf-D-HL(x) materials led to decreasing HMF conversion and increasing BBMF yield (Table 2). Although the amounts of B and L acid sites decreased with increasing x, S_micro_/S_EM_ decreased, favouring BBMF formation (Supplementary Figure S13B). Steric effects may be important for materials with higher S_micro_/S_EM_, negatively impacting on the formation of the relatively bulky diether BBMF.

The effect of the Hf loading was studied for yHf-D-HL(0.15) (y = 0.28, 1.40 mmol_Hf_ g^−1^) and yHf-D-HL(0.28) (y = 0.28, 1.12) (Table 2). A higher Hf loading did not enhance HMF conversion, total product yields or BBMF yields. No clear relationship could be established between the L acid strength of all the Hf-containing catalysts and BBMF yields. These results did not parallel those for the integrated acid-CTH reactions of LA. According to the literature, the optimal material properties (*e.g.*, L acidity) of Hf-zeolites for CTH reaction systems may depend on the type of substrate (Luo et al., 2014). HMF is a relatively bulky molecule and somewhat less reactive than LA and AnL, which may pose different requirements on materials properties.

## 4 Conclusion

Hierarchical multifunctional LTL zeotypes were prepared for the chemical valorization of HMF, LA and AnL via integrated catalytic transfer hydrogenation (CTH) and acid reactions in 2-butanol (2BuOH) at 200°C. This is the first CTH application reported for LTL related materials.

The catalysts were prepared via top-down strategies involving desilication, dealumination (x = 0.10–0.28 M H_2_SO_4_) and solid-state impregnation (SSI) of different amounts of hafnium (y), giving yHf-D-HL(x). The influence of the Hf loading (y) was studied for two groups of materials yHf-D-HL(x) with x = 0.15 (crystalline micro/mesoporous) and x = 0.28 (mostly amorphous, mesoporous).

Molecular level spectroscopic studies indicated: broader distributions of Al_tetra_ sites with increasing x; SSI/calcination introduced Lewis acid Hf sites; and the Hf loading (y) may affect, to different extents, the textural properties, distributions and types (and strength) of acid sites, *etc.* Mechanistic and kinetic studies suggested that Al sites may promote esterification and etherification reactions (*e.g.*, LA to 2BL), whereas Hf sites were required for CTH (*e.g.*, GVL formation from LA and AnL).

For materials with y = 0.28 mmol_Hf_ g^−1^ (lower Hf loading), GVL yields increased with decreasing Lewis acid strength and S_micro_/S_EM_ ratio. The decreasing L acid strength may avoid strongly adsorbed carbonaceous matter on the catalysts, and, on the other hand, lower S_micro_/S_EM_ may avoid steric hindrance and facilitate cyclization of intermediates (especially of relatively bulky ones, *e.g.*, esters versus corresponding carboxylic acids), enhancing GVL formation. For materials with y > 0.28 mmol_Hf_ g^−1^, GVL formation may depend on an interplay of several factors, *e.g.*, L acid strength, distribution and type of acid sites.

Although 0.28Hf-D-HL(0.28) was mostly amorphous and mesoporous, it performed far superiorly to ordered mesoporous hafnium silicate Hf-TUD-1 possessing similar Hf loading. The top-down strategy and, on the other hand, having an LTL zeolite in its genesis, may result in unique surface properties of yHf-D-HL(0.28) type materials. The 1.12Hf-D-HL(0.28) catalyst led to 77% GVL yield at 200°C/24 h (using 0.45 M initial LA concentration), and 91% GVL yield at 200°C/5 h (0.11 M initial LA concentration).

HMF was a more demanding substrate than LA and AnL for the formation of GVL. The main product was BMF, and GVL was not formed in measurable amounts at 200°C/24 h. HMF conversion to BMF and 2BL did not require Hf sites (HL was effective), whereas the diether BBMF was solely formed in the presence of Hf-containing catalysts (21% yield for 0.28Hf-D-HL(0.28)).

Based on catalytic and characterization studies, the materials were relatively stable. The zirconium catalyst 0.28Zr-D-HL(0.15) possessed acid and CTH activity, but performed inferiorly to the analogue 0.28Hf-D-HL(0.15).

Overall, post-synthesis modifications of commercial LTL zeolites may broaden their catalytic application profiles. The top-down strategies and conditions may be optimized to tune material properties and meet superior catalytic performances. A challenge may be to further enhance mesoporosity without considerable reduction of crystallinity.

## Data Availability

The original contributions presented in the study are included in the article/Supplementary Material, further inquiries can be directed to the corresponding authors.
