# Supplementary material for: Micro/mesoporous LTL derived materials for catalytic transfer hydrogenation and acid reactions of bio-based levulinic acid and furanics
Source: Front Chem. 2022 Sep 29;10:1006981. doi: 10.3389/fchem.2022.1006981 (PMC9558274; doi:10.3389/fchem.2022.1006981)
Supplement: Supplementary file 1 [file DataSheet1.docx]

Supplementary Material

# Introduction - A literature survey for LTL zeolitic materials reported for hydrogenation reactions using external supply of H_2_

A literature survey indicated that LTL zelitic catalysts were effective for hydrogenation reactions using external supply of H_2_, *e.g.*: (i) hydrogenation of phenol (Talukdar et al., 1993; Liu et al., 2021, 2022), citral (Álvarez-Rodríguez et al., 2005, 2006, 2008b, 2009, 2011, 2012), benzene (Simon et al., 2001) and diacetone alcohol (Liu et al., 1992), and hydrogenolysis of glycerol (Gallegos-Suarez et al., 2015), propane and butane (Weitkamp et al., 1994; Bond and Lin, 1997; Sho et al., 1999), in the presence of LTL zeolites impregnated with noble metals; (ii) hydrogenation of citral (Álvarez-Rodríguez et al., 2008a) and carbon monoxide (Cagnoli et al., 2002; Wang et al., 2005), in the presence of LTL zeolites possessing non-noble transition metals (*e.g.*, Ni, Cu); (iii) hydrogenation of xylose to xylitol in the presence of hierarchical K-LTL mechanically mixed with Pd/Al_2_O_3_ (Tangale et al., 2019).

# Experimental

## Materials

All reagents and solvents were obtained from commercial sources and used as received. For materials syntheses: hafnium(IV) acetylacetonate (Hf(acac)_4_, 97 %, Alfa-Aesar), zirconium(IV) acetylacetonate (Zr(acac)_4_, 98 %, Sigma-Aldrich), oxalic acid (98 %, Acros Organics), hafnium oxide HfO_2_ (99.95 %, metal basis excluding Zr, Alfa-Aesar), Sulfuric acid (Sigma-Aldrich, puriss p.a., ACS reagent, reag. ISO, reag. Ph. Eur., 95-97 %), potassium hydroxide (KOH, practical grade (P.A), eKa), ammonium nitrate (NH_4_NO_3_, 98%, Aldrich). KL zeolite was supplied by UOP (United States). For catalytic studies: levulinic acid (Aldrich, 98 %), α-angelica lactone (Alfa Aesar, 98 %), 5-(hydroxymethyl)furfural (Shangai, China), 2-butanol (2BuOH, Aldrich, puriss, p.a., Reag. Ph. Eur., ≥ 99.5 %), milli-Q water.

## Characterization of the materials

The X-ray powder diffraction (PXRD) data were collected on an Empyrean PANalytical diffractometer (Cu-Kα X-radiation, λ = 1.54060 Å) in a Bragg-Brentano *para*-focusing optics configuration (45 kV, 40 mA) at ambient temperature. Samples were prepared in a spinning flat plate sample holder and step-scanned in the range from 3° to 68° (2θ) with steps of 0.026°. A PIXEL linear detector with an active area of 1.7462° was used with a counting time of 68 s per step. The relative crystallinity was calculated from the PXRD patterns (subtracting artifactual background effects), considering the sum of the areas of the peaks of the LTL crystalline phase in relation to the total area in the range 3° to 68° (2θ) using the software HighScore. The same software (HighScore) was used to calculate the average crystallite sizes. Scanning electron microscopy (SEM) images, elemental mappings (Hf, Zr, Si, Al) and energy dispersive X-ray spectroscopy (EDS) analyses were obtained on a Hitachi SU-70 SEM microscope with a Bruker Quantax 400 detector operating at 20 kV. Nitrogen sorption isotherms were measured at -196 °C, using a Quantachrome instrument (automated gas sorption data using Autosorb IQ_2_). The samples were pre-treated at 250 °C for 3 h, under vacuum (< 4 × 10^-3^ bar). The specific surface area was calculated using the Brunauer, Emmett, Teller equation (S_BET_); external/mesoporous surface area (S_EM_) and micropore volume (V_micro_) were calculated using the t-plot method (micropore specific surface area, S_micro_ = S_BET_ - S_EM_); pore size distributions were determined by the DFT method (adsorption branch).

The ^27^Al MAS NMR spectra were recorded at 182.432 MHz using a Bruker Avance IIITM HD 700 MHz (16.4 T) spectrometer with a unique pulse, a recycle delay of 0.5 s and a spinning rate of 15 kHz; chemical shifts are quoted in ppm from Al(NO_3_)_3_. Attenuated Total Reflectance Fourier Transform Infrared (ATR FTIR) spectra were measured in transmittance mode using a Unican Mattson 7000 spectrometer equipped with a Specac Golden Gate Mk II ATR accessory having a diamond top-plate and KRS-5 focusing lenses (400-4000 cm^-1^, 256 scans, 4 cm^-1^ resolution). Elemental analysis for C was performed on a Leco TruSpec 630-200-200 analyzer.

The acid properties were measured using a NexusThermo Nicolet apparatus (64 scans and resolution of 4 cm^-1^) equipped with a specially designed cell, using self-supported discs (5–10 mg cm^-2^) and pyridine as base probe. After *in situ* outgassing at 450 °C for 3 h under vacuum (10^-6^ mbar), pyridine (99.99 %) was contacted with the sample at 150 °C for 10 min, and subsequently evacuated at 150 and 350 °C for 30 min, under vacuum. The quantitative measurements were based on the areas of the bands at *ca.* 1540 and 1430-1460 cm^-1^ which are associated with pyridine adsorbed on Brønsted (B) and Lewis (L) acid sites, respectively, and using Poitiers´ extinction molar coefficients (Morin et al., 1998). The L and B acid strengths were based on the molar ratio of acid sites measured after evacuation at 150 and 350 °C (L_350_/L_150_ and B_350_/B_150_, respectively); acid sites of moderate strength remain adsorbed at 150 °C, and relatively strong acid sites remain adsorbed at 350 °C.

## Treatment of D-KL using 0.4 M H_2_SO_4_

According to the literature, KL may tolerate 0.4 M H_2_SO_4_ treatment for 1 h (Al-Ani et al., 2019); these conditions were applied to D-KL, albeit this resulted in the complete loss of crystallinity (Figure S1).

**
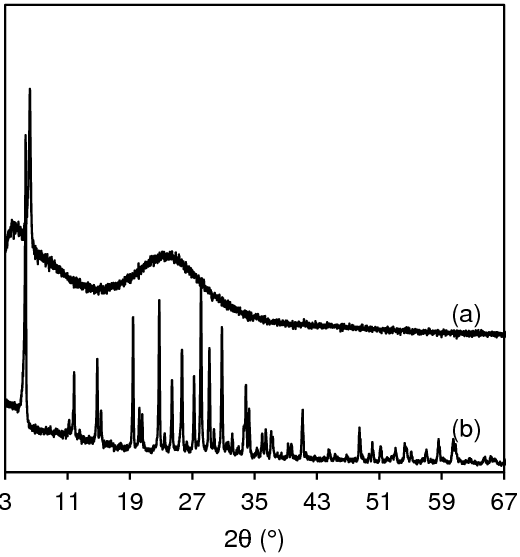
**

**Figure S1.** PXRD pattern of (a) D-KL treated with 0.4 M H_2_SO_4_ (1 h, room temperature), and for comparison, (b) commercial KL.

## Treatment of KL with NH_4_NO_3_

The parent zeolite KL was ion-exchanged three times using 0.15 M aq. NH_4_NO_3_ (40 mL per gram of zeolite) at 100 ºC, under reflux for 1 h. The solid was washed with Milli-Q water after each ion-exchange, and after the third ion-exchange the solid was washed, dried overnight at 80 ºC, and calcined at 550 ºC (1 ºC min^–1^) under air flow (20 mL min^-1^) for 6 h (Bartolomeu et al., 2016). However, crystalline phase contamination was verified via PXRD (Figure S2), which may be ammonium aluminum silicate and/or hydrogen potassium aluminum silicate hydrate. Hence, this material was not further explored.

| **Symbol** | **Ref. Code** | **Compound Name** |  | **Chemical Formula** |
| --- | --- | --- | --- | --- |
| Blue squares | 01-084-7371 | Ammonium Aluminum Silicate |  | (NH_4_)_0.94_Al_0.94_ Si_2.06_O_6_ |
| Green circles | 04-011-0705 | Hydrogen Potassium Aluminum Silicate Hydrate |  | H_4.36_K_5.14_Al_9.5_Si_26.5_ O_72_(H_2_O)_5.1_ |

**Figure S2.** PXRD pattern of KL treated with NH_4_NO_3_ (red) showing new peaks.

# Material Characterization Results

## 0.28Zr-D-HL(0.15)

Material 0.28Zr-D-HL(0.15) was characterized in a similar fashion to the Hf counterpart 0.28Hf-D-HL(0.15). This material possessed Si/Al = 8 and 0.28 mmol_Zr_ g^-1^ (equivalent to that for the hafnium analogue 0.28Hf-D-HL(0.15)), Table S1. The material exhibited reflections characteristic of the LTL topology (Figure S3A). The metals distributions were uniform according to the elemental (Si, Al, Zr) mapping images (Figure S3C). Zr-D-HL(0.15) possessed S_micro_, S_EM_ and V_micro_ of 92 m^2^ g^-1^, 120 m^2^ g^-1^, 0.05 cm^3^ g^-1^, respectively (Table S2). The ^27^Al MAS NMR spectrum showed a main resonance at *ca*. 63 ppm due to Al_tetra_ and a very small resonance at *ca*. 0 ppm due to Al_octa_ (Figure S3B).


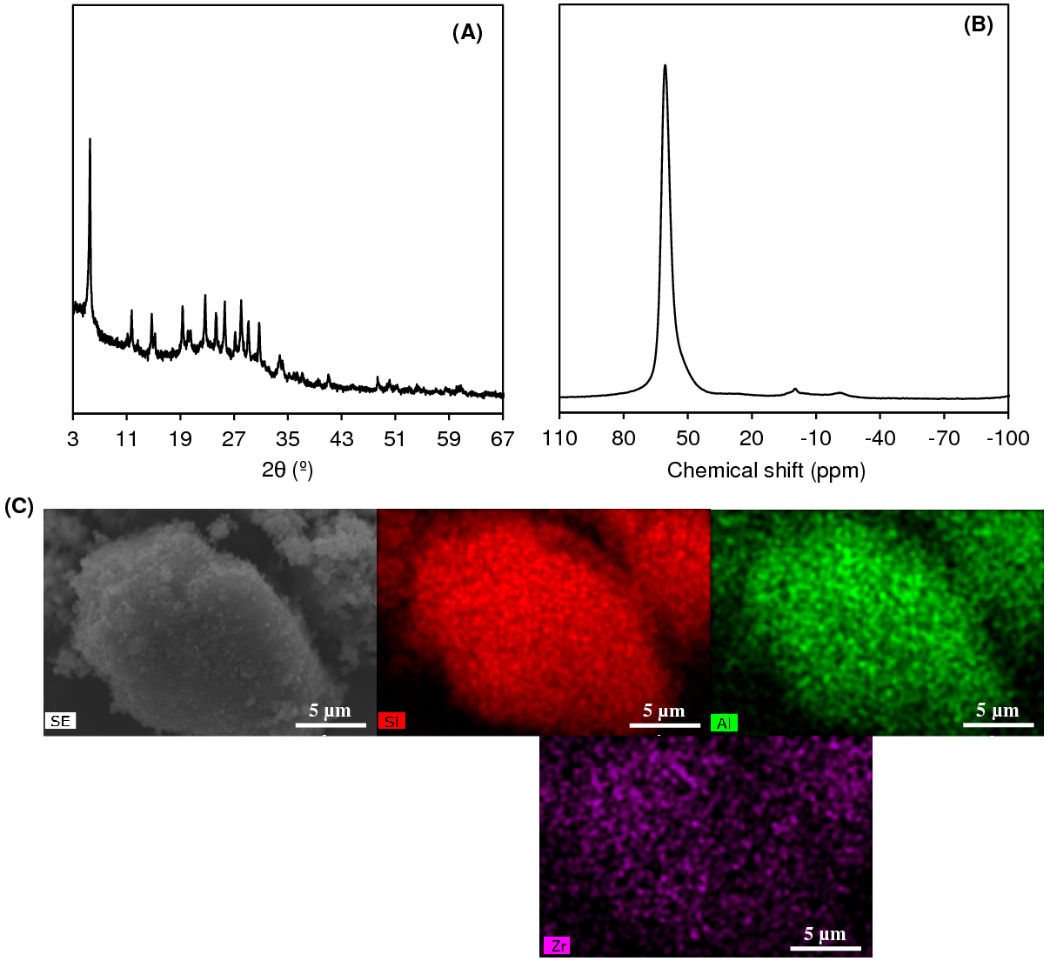


**Figure S3.** PXRD pattern (A), ^27^Al NMR spectrum (B) and SEM and mappings (C) of 0.28Zr-D-HL(0.15).

## Various materials prepared

**Table S1.** Composition and relative crystallinities (RC) of the prepared materials.

| **Sample** | **Si/Al** | **Hf**  **(mmol g^-1^)** | **Relative crystallinity**  **(RC, %)** |
| --- | --- | --- | --- |
| KL | 3 | - | - |
| KL(oxac) | 5 | - | 96 |
| D-KL | 3 | - | 99 |
| D-HL(0.10) | 5 | - | 87 |
| 0.28Hf-D-HL(0.10) | 5 | 0.28 | 82 |
| D-HL(0.15) | 9 | - | 73 |
| 028Hf-D-HL(0.15) | 7 | 0.28 | 71 |
| 0.56Hf-D-HL(0.15) | 7 | 0.56 | 57 |
| 0.84Hf-D-HL(0.15) | 8 | 0.84 | 50 |
| 1.12Hf-D-HL(0.15) | 7 | 1.12 | 41 |
| 1.40Hf-D-HL(0.15) | 8 | 1.40 | 38 |
| 1.68Hf-D-HL(0.15) | 7 | 1.68 | ^a^ |
| D-HL(0.20) | 16 | - | 36 |
| 0.28Hf-D-HL(0.20) | 16 | 0.28 | 22 |
| D-HL(0.28) | 41 | - | 22 |
| 0.28Hf-D-HL(0.28) | 41 | 0.28 | 20 |
| 0.56Hf-D-HL(0.28) | 40 | 0.56 | 24 |
| 0.84Hf-D-HL(0.28) | 39 | 0.84 | 21 |
| 1.12Hf-D-HL(0.28) | 40 | 1.12 | 18 |
| 1.40Hf-D-HL(0.28) | 43 | 1.40 | ^a^ |
| 1.68Hf-D-HL(0.28) | 41 | 1.68 | ^a^ |
| 0.28Zr-D-HL(0.15) | 8 | 1.40 | 64 |
| HL | 6 | - | - |
| 0.28Hf-HL | 6 | 0.28 | - |

^a^ The material possessed nanocrystalline hafnium.

**Table S2.** Textural properties of the materials prepared.

| **Material** | **S_BET_**  **(m^2^ g^-1^)** | **S_EM_**  **(m^2^ g^-1^)** | **S_micro_**  **(m^2^ g^-1^)** | **S_micro_/S_EM_** | **V_micro_**  **(cm^3^ g^-1^)** |
| --- | --- | --- | --- | --- | --- |
| KL | 331 | 32 | 299 | 9.34 | 0.12 |
| KL(oxac) | 399 | 57 | 291 | 5.11 | 0.16 |
| D-KL | 479 | 134 | 345 | 2.57 | 0.15 |
| D-HL(0.10) | 463 | 132 | 331 | 2.51 | 0.15 |
| 0.28Hf-D-HL(0.10) | 265 | 112 | 153 | 1.37 | 0.07 |
| D-HL(0.15) | 350 | 130 | 220 | 1.69 | 0.10 |
| 0.28Hf-D-HL(0.15) | 242 | 125 | 117 | 0.94 | 0.07 |
| 1.40Hf-D-HL(0.15) | 217 | 138 | 79 | 0.57 | 0.04 |
| D-HL(0.20) | 270 | 148 | 122 | 0.82 | 0.05 |
| 0.28Hf-D-HL(0.20) | 230 | 134 | 96 | 0.72 | 0.04 |
| D-HL(0.28) | 128 | 117 | 11 | 0.09 | 0.01 |
| 0.28Hf-D-HL(0.28) | 119 | 111 | 8 | 0.07 | 0.01 |
| 1.12Hf-D-HL(0.28) | 96 | 74 | 22 | 0.30 | 0.01 |
| HL | 284 | 96 | 188 | 1.96 | 0.08 |
| 0.28Zr-D-HL(0.15) | 212 | 120 | 92 | 0.77 | 0.05 |

**
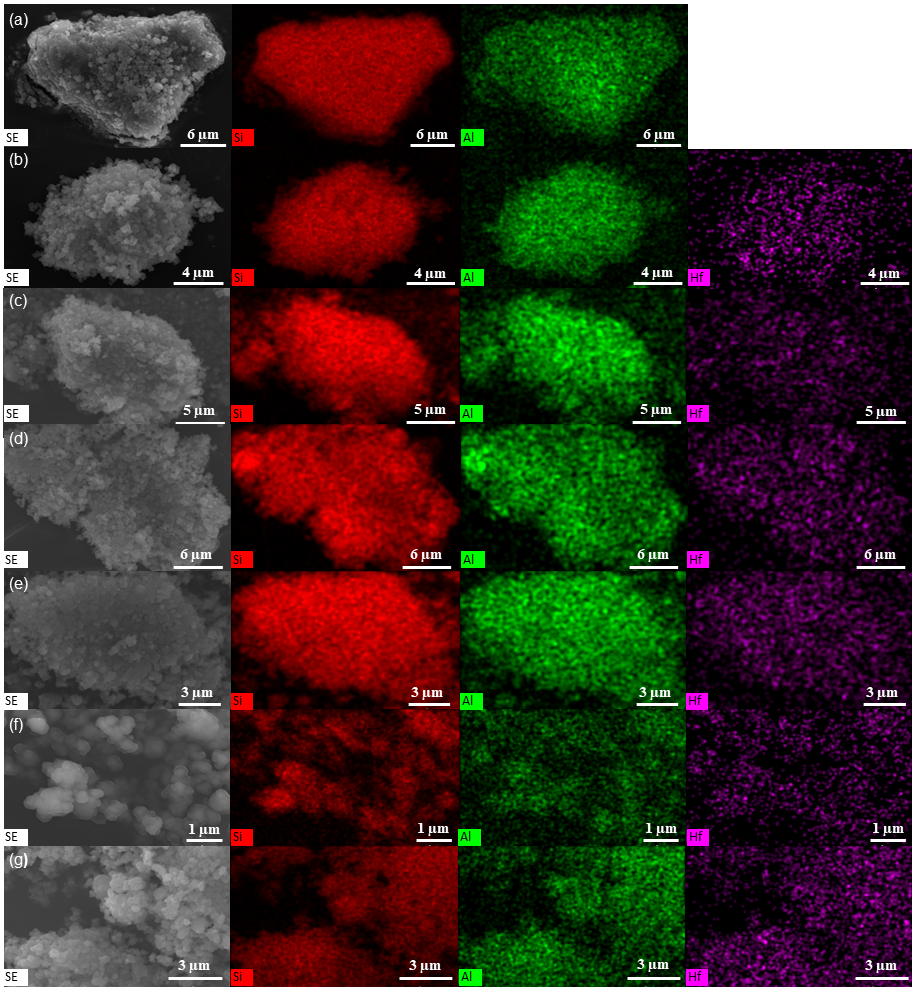
**

**Figure S4**. SEM images (left image per line) and element mappings (Si – red, Al – green, Hf – purple) of D-HL(0.15) (a) and yHf-D-HL(0.15) (y = 0.28 (b), 0.56 (c), 0.84 (d), 1.12 (e), 1.40 (f), 1.68 (g)).


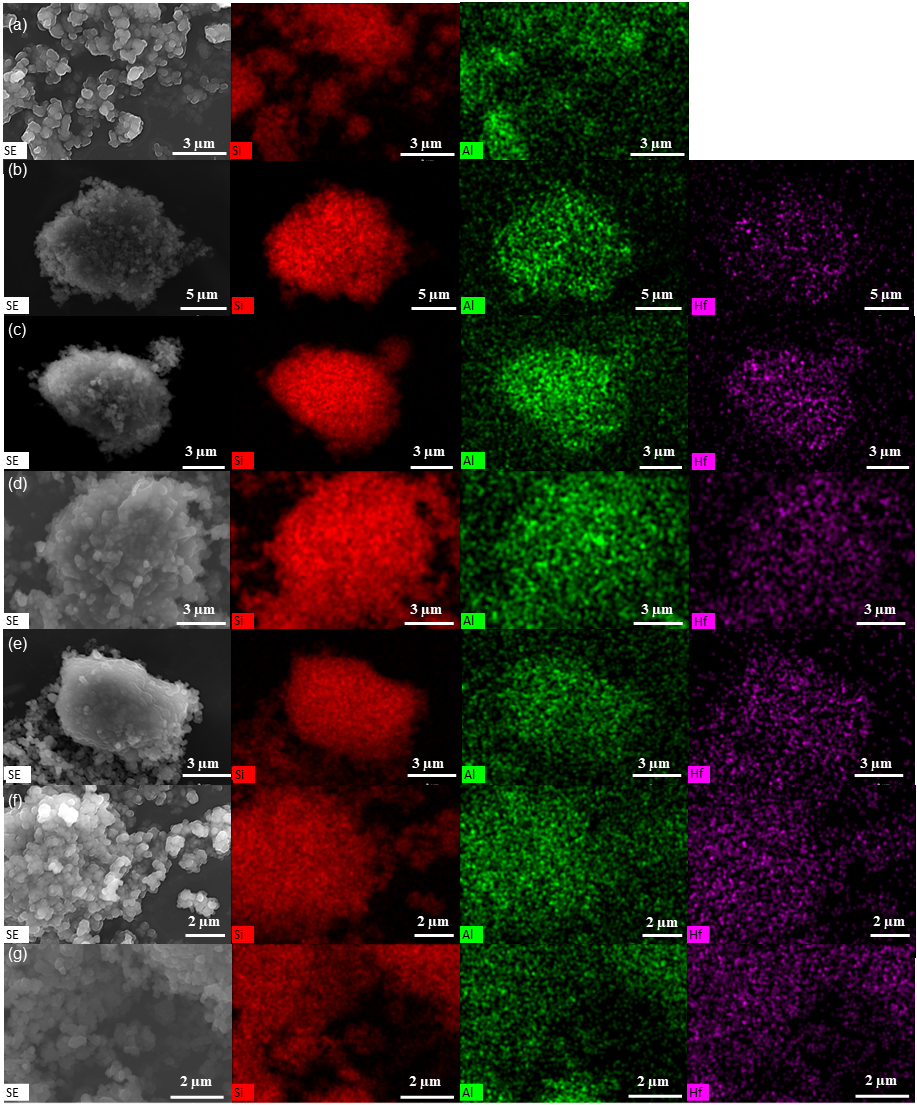


**Figure S5**. SEM images (left image per line) and element mappings (Si – red, Al – green, Hf – purple) of D-HL(0.28) (a) and yHf-D-HL(0.28) with y = 0.28 (b), 0.56 (c), 0.84 (d), 1.12 (e), 1.40 (f), 1.68 (g).


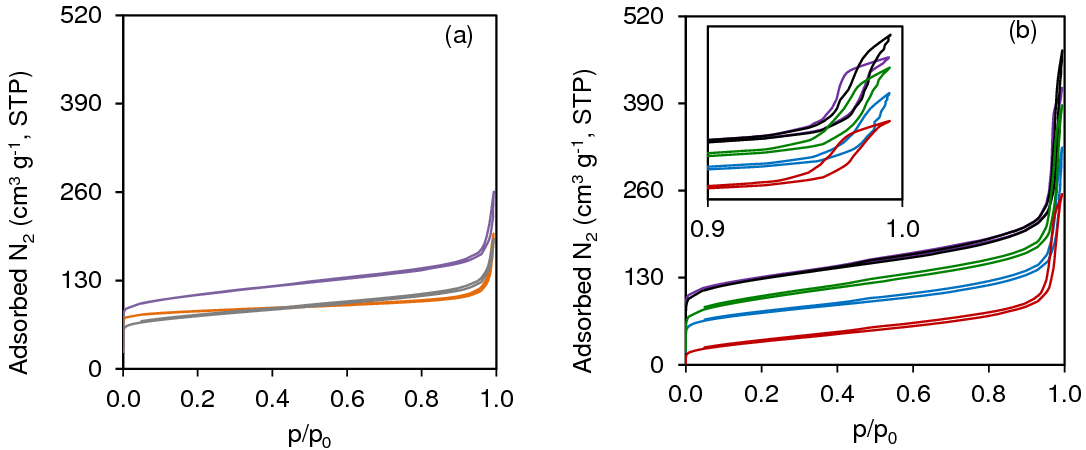


**Figure S6.** Nitrogen sorption isotherms at -196 ºC of (a) KL (orange), HL (grey), D-KL (purple); and (b) D-KL (purple), D-HL(x) with x = 0.10 (black), 0.15 (blue), 0.20 (green), 0.28 (red).


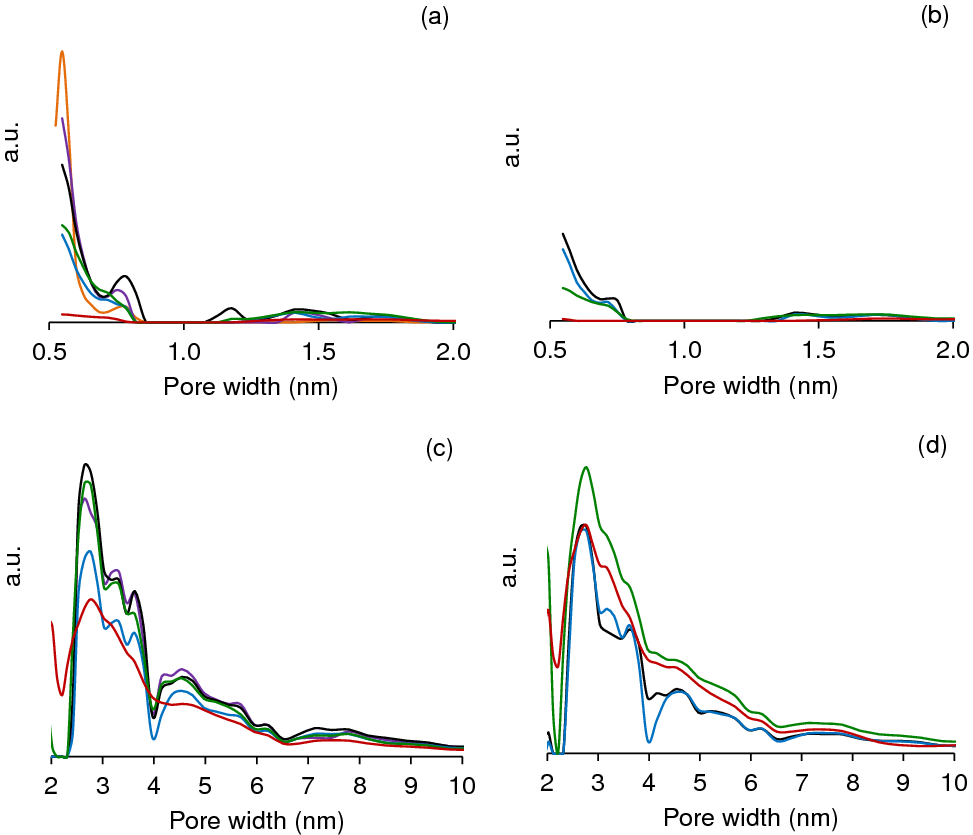


**Figure S7.** Micropore (a, b) and mesopore (c, d) size distributions of (a, c) KL (orange), D-KL (purple) and D-HL(x) with x = 0.10 (black), 0.15 (blue), 0.20 (green), 0.28 (red); and (b, d) 0.28Hf-D-HL(x) with x = 0.10 (black), 0.15 (blue), 0.20 (green), 0.28 (red).


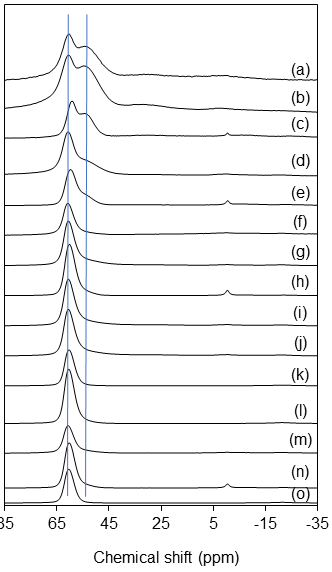


**Figure S8.** ^27^Al MAS NMR spectra of 1.12Hf-D-HL(0.28) (a), 0.28Hf-D-HL(0.28) (b), D-HL(0.28) (c), 0.28Hf-D-HL(0.20) (d), D-HL(0.20) (e), 1.40Hf-D-HL(0.15) (f), 0.28Hf-D-HL(0.15) (g), D-HL(0.15) (h), 0.28Hf-D-HL(0.10) (i), D-HL(0.10) (j), D-KL (k), KL(oxac) (l), 0.28Hf-HL (m), HL (n) and KL (o).

**Table S3.** Acid properties of the materials prepared.

| **Material** | **L**^a^  **(µmol g^-1^)** | **B**^a^  **(****µmol g^-1^)** | **L+B**^a^  **(µmol g^-1^)** | **L/B** | **L_350_/L_150_**^b^ |
| --- | --- | --- | --- | --- | --- |
| 0.28Hf-D-HL(0.10) | 216 | 80 | 296 | 2.7 | 0.33 |
| 0.28Hf-D-HL(0.15) | 91 | 53 | 144 | 1.7 | 0.32 |
| 0.56Hf-D-HL(0.15) | 149 | 75 | 224 | 2.0 | 0.26 |
| 0.84Hf-D-HL(0.15) | 146 | 59 | 205 | 2.5 | 0.25 |
| 1.12Hf-D-HL(0.15) | 128 | 61 | 189 | 2.1 | 0.25 |
| 1.40Hf-D-HL(0.15) | 115 | 60 | 175 | 1.9 | 0.23 |
| 0.28Hf-D-HL(0.20) | 54 | 23 | 77 | 2.3 | 0.17 |
| 0.28Hf-D-HL(0.28) | 33 | 14 | 47 | 2.4 | 0.15 |
| 0.56Hf-D-HL(0.28) | 42 | 16 | 58 | 2.6 | 0.12 |
| 0.84Hf-D-HL(0.28) | 60 | 13 | 73 | 4.6 | 0.08 |
| 1.12Hf-D-HL(0.28) | 56 | 11 | 67 | 5.1 | 0.04 |
| 1.40Hf-D-HL(0.28) | 54 | 6 | 60 | 9.0 | 0.09 |
| HL | 82 | 45 | 127 | 1.8 | 0.19 |
| 0.28Hf-HL | 128 | 71 | 199 | 1.8 | 0.39 |

^a^ Measured by FT-IR spectroscopy of adsorbed pyridine (150 ºC): L = Lewis acid sites; B = Brønsted acid sites. ^b^ Molar ratio L acid sites measured at 350 ºC (L_350_) and 150 ºC (L_150_); B_350_/B_150_ ≅ 0.

# Catalytic Results


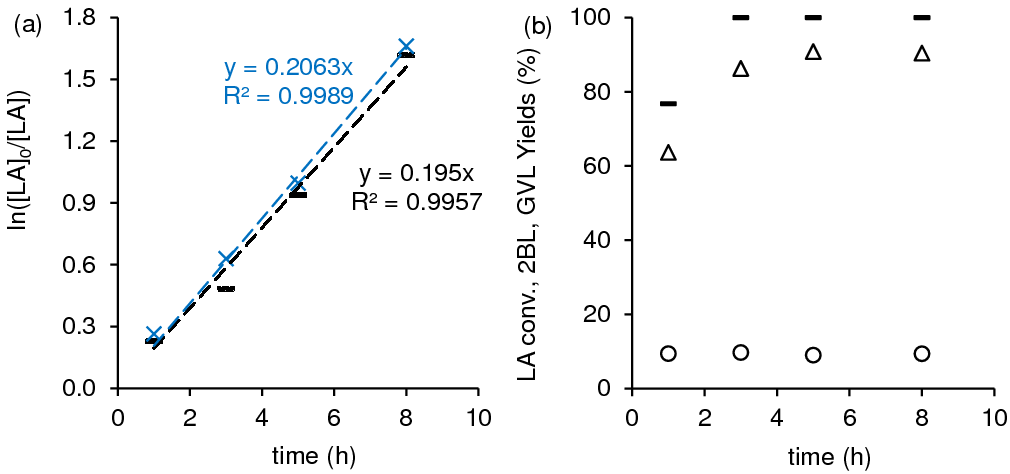


**Figure S9**. (a) Dependency of ln([LA]_0_/[LA]) on reaction time for LA conversion in the presence of 1.40Hf-D-HL(0.15) (+) or 1.12Hf-D-HL(0.28) (×); integrated rate law, ln([LA]_0_/[LA]) = *k* t, where *k* is the kinetic constant and *t* is reaction time. Reaction conditions: 0.45 M LA in 2BuOH, 25.5 g_cat_ L^-1^, 200 ºC, 24 h. (b) Dependency of LA conversion (-), GVL yield (Δ) and 2BL yield (o) on reaction time for 1.12Hf-D-HL(0.28). Reaction conditions: 0.11 M LA in 2BuOH, 25.5 g_cat_ L^-1^, 200 ºC, 24 h.

**Table S4.** Comparison of the catalytic results for yHf-D-HL(x) to literature data for the reaction of LA to GVL, in the presence of micro/mesoporous zeotypes or mesoporous silicas/silicates, using a secondary alcohol (ROH) as *in situ* H-donor.^a^

| **Entry** | **Catalyst** | **T (**º**C)** | **t (h)** | **ROH** | **[LA]_0_**  **(M)** | **Cat (wt)** | **X**  **(%)** | **Y_GVL_**  **(%)** | **Ref** |
| --- | --- | --- | --- | --- | --- | --- | --- | --- | --- |
| 1 | 0.28Zr-D-H(0.15) (5wt% Hf) | 200 | 24 | 2BuOH | 0.45 | 0.5 | 96 | 10 | - |
| 2 | 0.28Hf-D-HL(0.15) (5wt% Hf) | 200 | 24 | 2BuOH | 0.45 | 0.5 | 100 | 23 | - |
| 3 | 1.40Hf-D-HL(0.15) (20wt% Hf) | 200 | 24/5 | 2BuOH | 0.45/0.11 | 0.5/1.95 | 98/100 | 38/81 | - |
| 4 | 0.28Hf-D-H(0.28) (5wt% Hf) | 200 | 24 | 2BuOH | 0.45 | 0.5 | 100 | 51 | - |
| 5 | 1.12Hf-D-HL(0.28) (15wt% Hf) | 200 | 24/5 | 2BuOH | 0.45/0.11 | 0.5/1.95 | 100/100 | 77/91 | - |
| 6 | Hf-TUD-1 (Si/Hf=50) | 200 | 24 | 2BuOH | 0.45 | 0.5 | 100 | 29 | - |
| 7 | Hf(4.8wt%)WdeSAlBeta-m | 180 | 24 | 2BuOH | 0.45 | 0.5 | 100 | 99 | (Antunes et al., 2022) |
| 8 | (Sn)_SSIE_-beta1 (Si/Sn=20) | 120 | 24/72 | 2BuOH | 0.45 | 0.51 | 44/68 | 6/25 | (Antunes et al., 2015) |
| 9 | SnAl-Beta (2.7wt% Sn) | 160 | 6 | 2PrOH | 0.11 | 0.63 | 73 | 53 | (Winoto et al., 2016) |
| 10 | (Zr)_SSIE_-beta (Si/Zr=17) | 120 | 24 | 2BuOH | 0.45 | 0.51 | 56 | 22 | (Antunes et al., 2016a) |
| 11 | Zr-AlBeta (Si/Zr=32) | 170 | 6 | 2PrOH | 0.15 | 0.69 | 100 | 92 | (López-Aguado et al., 2020) |
| 12 | Zr-AlBeta (Si/Zr=32) | 170 | 6 | 2PrOH | 0.16 | 0.67 | 100 | 87 | (Morales et al., 2019) |
| 13 | Zr-AlBeta/TUD-1 composite | 150 | 24/72 | 2BuOH | 0.45 | 0.51 | 63/91 | 6/31 | (Antunes et al., 2016b) |
| 14 | Zr-TUD-1 (Si/Zr=28) | 120 | 24 | 2BuOH | 0.45 | 0.51 | 32 | - | (Antunes et al., 2016a) |
| 15 | ZrAl-TUD-1 (Si/Zr=39) | 120 | 24 | 2BuOH | 0.45 | 0.51 | 42 | - | (Antunes et al., 2016a) |
| 16 | CePO_4_(40wt%)@H-Beta | 140 | 24 | 2PrOH | 0.17 | 0.86 | 63 | 35 | (Kumar and Srivastava, 2019) |
| 17 | Hf(2wt%)-USY | 150 | 10 | 2PrOH | 0.20 | 0.86 | 99 | 95 | (Tang et al., 2019) |
| 18 | Zr(7.6wt%)-SBA-15-SGD^b^ | 160 | 6 | 2BuOH^c^ | 0.2 | 1.72 | 55 | 33 | (Ostovar et al., 2021) |
| 19 | Zr(20wt%)-SBA-15 | 160 | 18 | 2BuOH | 0.17 | 2.07 | >99 | 95 | (Zhou et al., 2018) |
| 20 | ZrO_2_(10wt%)/SBA-15 | 150 | 3 | 2PrOH^c^ | 0.2 | 0.17 | 100 | 90 | (Kuwahara et al., 2017) |
| 21 | ZrO_2_(10wt%)/SBA-15 | 150 | 2 | 2PrOH^c^ | 0.2 | 0.17 | 99 | 83 | (Kuwahara et al., 2014) |
| 22 | Sn(25wt%)-SBA-15 | 200 | 3 | 2PrOH | 0.04 | 0.26 | 72 | 71 | (Kumaravel et al., 2020) |
| 23 | SnO_2_(3.7wt%)-SBA-15 | 110 | 8 | 2PrOH | 0.79 | 0.40 | 85 | 81 | (Xu et al., 2017) |
| 24 | Zr(10wt%)-KIT-5 | 180 | 6 | 2PrOH | 0.20 | 0.86 | 97 | 89 | (He et al., 2020) |
| 25 | Ni(2wt%)-Sepiolite | 180 | 2 | 2PrOH | 0.35 | 1.22 | 25 | <1 | (García et al., 2021) |

^a^ Catalytic reaction temperature (T) and time (t), secondary alcohol used as reducing agent (ROH), wt% catalyst (based on initial mass of LA), LA conversion (X) and GVL yield (Y_GVL_). ^b^ SGD = sulfaguanidine surface groups. ^c^ Reaction carried out under 10 bar.

# Characterization of used catalysts


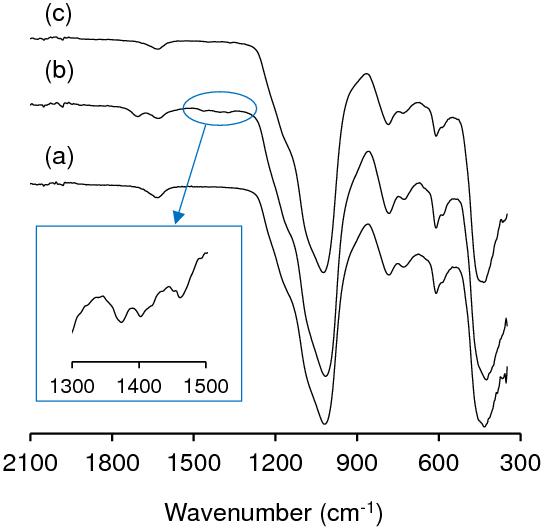


**Figure S10.** ATR FT-IR spectra of fresh 0.28Hf-D-HL(0.15) (a) and the respective used washed-dried (b) and washed-dried-calcined (c) solids. Reaction conditions: 0.45 M LA in 2BuOH, 200 ºC, 5 h.


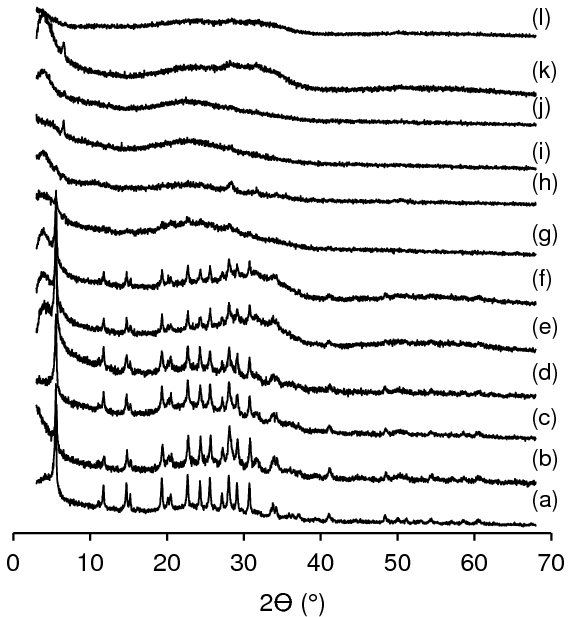


**Figure S11.** PXRD patterns of fresh (a) and used (b) 0.28Hf-D-HL(0.10); fresh (c) and used (d) 0.28Hf-D-HL(0.15); fresh (e) and used (f) 1.40Hf-D-HL(0.15); fresh (g) and used (h) 0.28Hf-D-HL(0.20); fresh (i) and used (j) 0.28Hf-D-HL(0.28); fresh (k) and used (l) 1.12Hf-D-HL(0.28).

**Table S5.** Textural and acid properties of the fresh and used catalysts 1.40Hf-D-HL(0.15) and 1.12Hf-D-HL(0.28).

| **Sample** | **S_micro_**  **(m^2^ g^-1^)** | **S_EM_**  **(m^2^ g^-1^)** | **V_micro_**  **(cm^3^ g^-1^)** | **Amount of acid sites** ^a^  **(µmol g^-1^)** | | | | **L_350_/L_150_** |
| --- | --- | --- | --- | --- | --- | --- | --- | --- |
|  |  |  |  | **L** | **B** | | **L+B** |  |
| 1.40Hf-D-HL(0.15) | 79 | 138 | 0.04 | 115 | 60 | 175 | | 0.23 |
| 1.40Hf-D-HL(0.15) used | 75 | 119 | 0.04 | 107 | 52 | 159 | | 0.21 |
| 1.12Hf-D-HL(0.28) | 22 | 74 | 0.01 | 56 | 11 | 67 | | 0.04 |
| 1.12Hf-D-HL(0.28) used | 15 | 86 | 0.01 | 60 | 3 | 63 | | 0.02 |

^a^ Measured by FT-IR spectroscopy of adsorbed pyridine (150 ºC): L = Lewis acid sites; B = Brønsted acid sites.


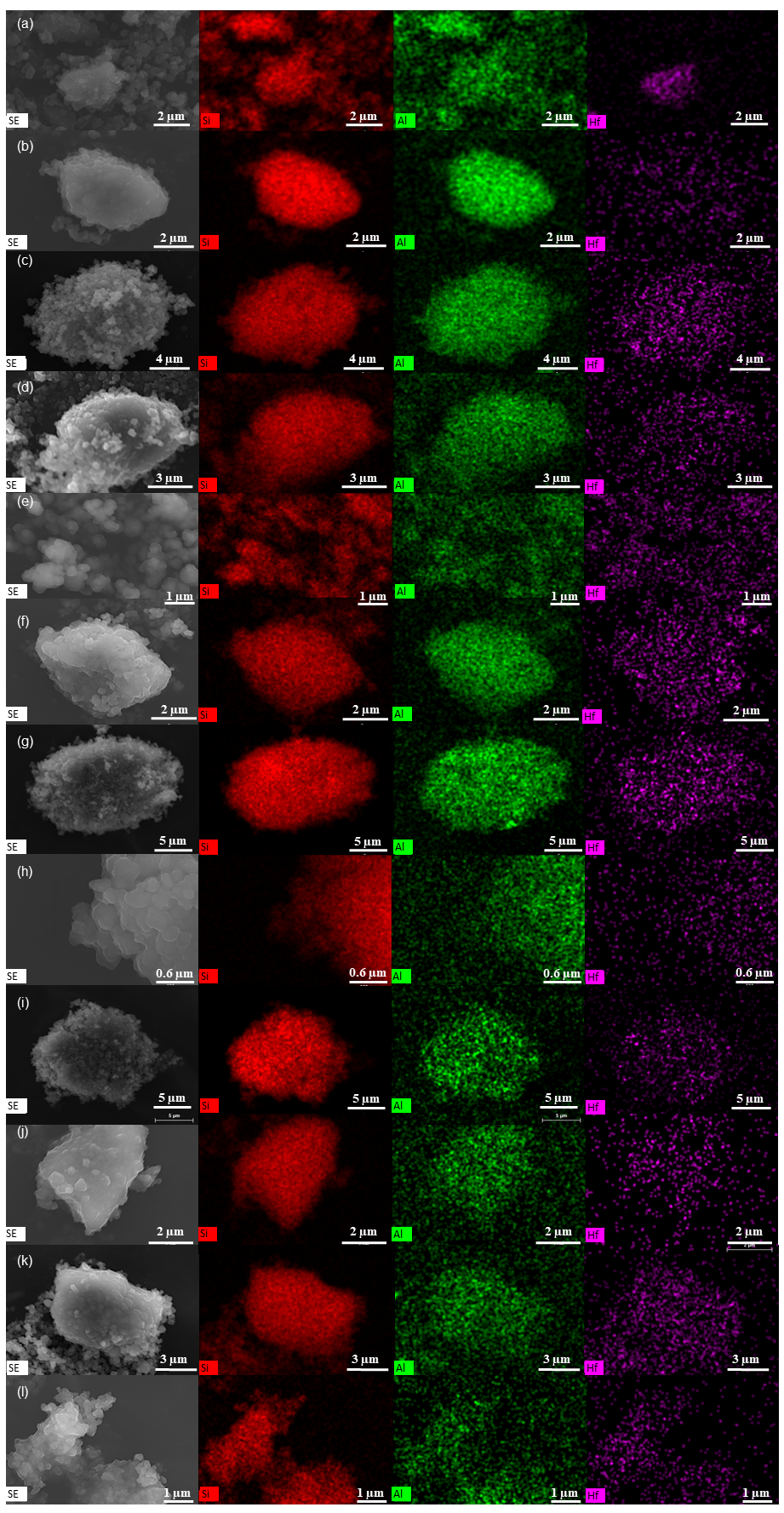


**Figure S12**. SEM images and elemental mappings (Si = red, Al = green, Hf = purple) of fresh (a) and used (b) 0.28Hf-D-HL(0.10); fresh (c) and used (d) 0.28Hf-D-HL(0.15); fresh (e) and used (f) 1.40Hf-D-HL(0.15); fresh (g) and used (h) 0.28Hf-D-HL(0.20); fresh (i) and used (j) 0.28Hf-D-HL(0.28); and fresh (k) and used (l) 1.12Hf-D-HL(0.28).


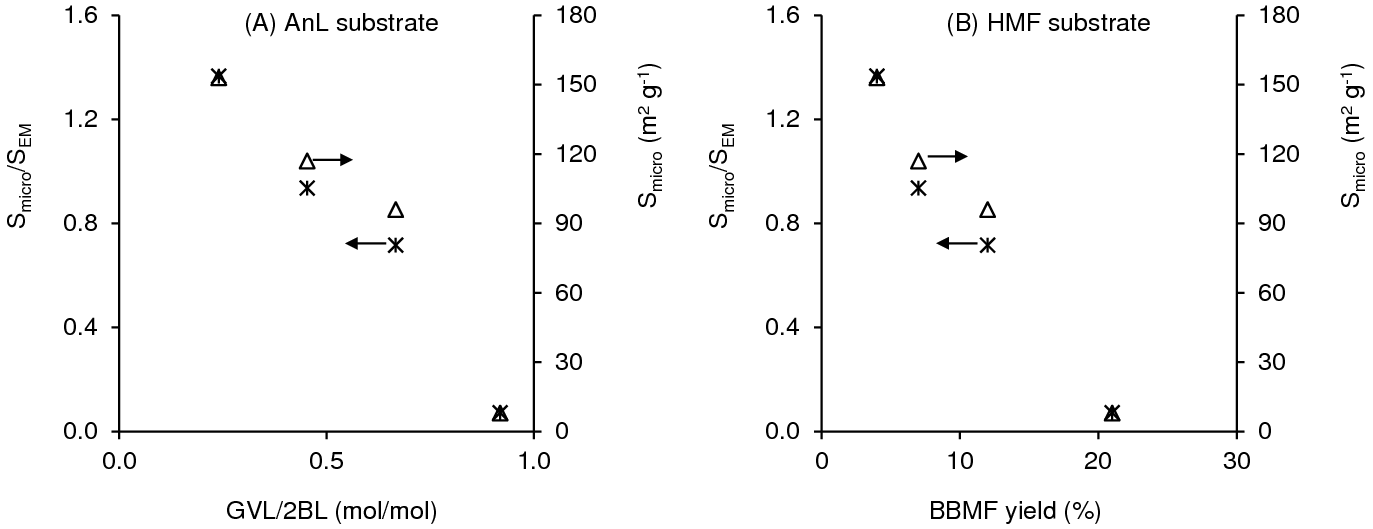


**Figure S13.** Relation between the textural properties of 0.28Hf-D-HL(x) and the GVL/2BL molar ratios for the reaction of α-angelica lactone (AnL) (A); similar trends were verified for GVL yields (instead of GVL/2BL) with each substrate. (B) Relation between the textural properties of 0.28Hf-D-HL(x) and BBMF yields, for the reaction of HMF. Reaction conditions: 0.45 M substrate in 2BuOH, 25.5 g_cat_ L^-1^, 200 ºC, 24 h.

# References

Al-Ani, A., Haslam, J. J. C., Mordvinova, N. E., Lebedev, O. I., Vicente, A., Fernandez, C., et al. (2019). Synthesis of nanostructured catalysts by surfactant-templating of large-pore zeolites. *Nanoscale Adv.* 1, 2029–2039. doi: 10.1039/c9na00004f.

Álvarez-Rodríguez, J., Cerro-Alarcón, M., Guerrero-Ruiz, A., Rodríguez-Ramos, I., and Arcoya, A. (2008a). Effect of nickel precursor and the copper addition on the surface properties of Ni/KL-supported catalysts for selective hydrogenation of citral. *Appl. Catal. A: Gen.* 348, 241–250. doi: 10.1016/j.apcata.2008.07.001.

Álvarez-Rodríguez, J., Guerrero-Ruiz, A., Rodríguez-Ramos, I., and Arcoya-Martín, A. (2005). Modifications of the citral hydrogenation selectivities over Ru/KL-zeolite catalysts induced by the metal precursors. in *Catalysis Today*, 302–309. doi: 10.1016/j.cattod.2005.07.088.

Álvarez-Rodríguez, J., Guerrero-Ruiz, A., Rodríguez-Ramos, I., and Arcoya-Martín, A. (2006). Surface and structural effects in the hydrogenation of citral over RuCu/KL catalysts. *Microporous Mesoporous Mater.* 97, 122–131. doi: 10.1016/j.micromeso.2006.07.016.

Álvarez-Rodríguez, J., Guerrero-Ruiz, A., Rodríguez-Ramos, I., and Arcoya, A. (2008b). Changes in the selective hydrogenation of citral induced by copper addition to Ru/KL catalysts. *Microporous Mesoporous Mater.* 110, 186–196. doi: 10.1016/j.micromeso.2007.06.006.

Álvarez-Rodríguez, J., Rodríguez-Ramos, I., Guerrero-Ruiz, A., and Arcoya, A. (2009). Surface changes in Ru/KL supported catalysts induced by the preparation method and their effect on the selective hydrogenation of citral. *Appl. Catal. A: Gen.* 366, 114–121. doi: 10.1016/j.apcata.2009.06.038.

Álvarez-Rodríguez, J., Rodríguez-Ramos, I., Guerrero-Ruiz, A., and Arcoya, A. (2011). Selective hydrogenation of citral over Pt/KL type catalysts doped with Sr, La, Nd and Sm. *Appl. Catal. A: Gen.* 401, 56–64. doi: 10.1016/j.apcata.2011.04.045.

Álvarez-Rodríguez, J., Rodríguez-Ramos, I., Guerrero-Ruiz, A., Gallegos-Suarez, E., and Arcoya, A. (2012). Influence of the nature of support on Ru-supported catalysts for selective hydrogenation of citral. *Chem. Eng. J.* 206, 169–178. doi: 10.1016/j.cej.2012.07.099.

Antunes, M. M., Lima, S., Neves, P., Magalhães, A. L., Fazio, E., Fernandes, A., et al. (2015). One-pot conversion of furfural to useful bio-products in the presence of a Sn,Al-containing zeolite beta catalyst prepared via post-synthesis routes. *J. Catal.* 329, 522–537. doi: https://doi.org/10.1016/j.jcat.2015.05.022.

Antunes, M. M., Lima, S., Neves, P., Magalhães, A. L., Fazio, E., Neri, F., et al. (2016a). Integrated reduction and acid-catalysed conversion of furfural in alcohol medium using Zr,Al-containing ordered micro/mesoporous silicates. *Appl. Catal. B Environ.* 182, 485–503. doi: 10.1016/j.apcatb.2015.09.053.

Antunes, M. M., Neves, P., Fernandes, A., Lima, S., Silva, A. F., Ribeiro, M. F., et al. (2016b). Bulk and composite catalysts combining BEA topology and mesoporosity for the valorisation of furfural. *Catal. Sci. Technol.* 6, 7812–7829. doi: 10.1039/c6cy00223d.

Antunes, M. M., Silva, A. F., Fernandes, A., Pillinger, M., Ribeiro, F., and Valente, A. A. (2022). Renewable bio-based routes to γ-valerolactone in the presence of hafnium nanocrystalline or hierarchical microcrystalline zeotype catalysts. *J. Catal.* 406, 56–71. doi: 10.1016/j.jcat.2021.12.022.

Bartolomeu, R., Bértolo, R., Casale, S., Fernandes, A., Henriques, C., Costa, P. da, et al. (2016). Microporous and Mesoporous Materials. *Microporous Mesoporous Mater.*, 137–147. doi: 10.5772/61556.

Bond, G. C., and Lin, X. (1997). Hydrogenolysis of propane and of n-butane on Pt/KL zeolite. *J. Catal.* 169, 76–84. doi: 10.1006/jcat.1997.1688.

Cagnoli, M. V, Gallegos, N. G., Alvarez, A. M., Bengoa, J. F., Yeramián, A. A., Schmal, M., et al. (2002). Catalytic CO hydrogenation on potassic Fe/zeolite LTL. *Appl. Catal. A: Gen.* 230, 169–176. doi: 10.1016/S0926-860X(01)01012-2.

Gallegos-Suarez, E., Guerrero-Ruiz, A., Rodriguez-Ramos, I., and Arcoya, A. (2015). Comparative study of the hydrogenolysis of glycerol over Ru-based catalysts supported on activated carbon, graphite, carbon nanotubes and KL-zeolite. *Chem. Eng. J.* 262, 326–333. doi: 10.1016/j.cej.2014.09.121.

García, A., Miguel, P. J., Pico, M. P., Álvarez-Serrano, I., López, M. L., García, T., et al. (2021). γ-valerolactone from levulinic acid and its esters: Substrate and reaction media determine the optimal catalyst. *Appl. Catal. A: Gen.* 623, 118276. doi: doi.org/10.1016/j.apcata.2021.118276.

He, J., Li, H., Xu, Y., and Yang, S. (2020). Dual acidic mesoporous KIT silicates enable one-pot production of g -valerolactone from biomass derivatives via cascade reactions. *Renew. Energy* 146, 359–370. doi: 10.1016/j.renene.2019.06.105.

Kumar, A., and Srivastava, R. (2019). CePO4, a multi-functional catalyst for carbohydrate biomass conversion: Production of 5-hydroxymethylfurfural, 2,5-diformylfuran, and γ-valerolactone. *Sustain. Energy Fuels* 3, 2475–2489. doi: 10.1039/c9se00272c.

Kumaravel, S., Thiripuranthagan, S., Durai, M., Erusappan, E., and Vembuli, T. (2020). Catalytic transfer hydrogenation of biomass-derived levulinic acid to γ-valerolactone over Sn/Al-SBA-15 catalysts. *New J. Chem.* 44, 8209–8222. doi: 10.1039/d0nj01288b.

Kuwahara, Y., Kaburagi, W., and Fujitani, T. (2014). Catalytic Conversion of LevulinicAcid and Its Esters to γ-Valerolactone over Silica- Supported Zirconia Catalysts. *Bull. Chem. Soc. Jpn.* 87, 1252–1254. doi: doi.org/10.1246/bcsj.20140205.

Kuwahara, Y., Kaburagi, W., Osada, Y., Fujitani, T., and Yamashita, H. (2017). Catalytic transfer hydrogenation of biomass-derived levulinic acid and its esters to γ-valerolactone over ZrO2 catalyst supported on SBA-15 silica. *Catal. Today* 281, 418–428. doi: 10.1016/j.cattod.2016.05.016.

Liu, C., Wang, J., Zhu, P., Liu, H., and Zhang, X. (2022). Relating the performances of selective phenol hydrogenation with encapsulated palladium nanoparticles and surrounding distinct LTL-zeolite microenvironments. *Chem. Eng. J.* 430, 132589. doi: 10.1016/j.cej.2021.132589.

Liu, C., Zhu, P., Liu, H., and Zhang, X. (2021). Tailoring Locations and Electronic States of Rh Nanoparticles in KL Zeolite by Varying the Reduction Temperature for Selective Phenol Hydrogenation. *Ind. Eng. Chem. Res.* 60, 17489–17499. doi: 10.1021/acs.iecr.1c03653.

Liu, Y. Y., Zhao, W., Zhang, S., and Fang, Y. (1992). XPS and EDX study on an RuKL zeolite hydrogenation catalyst. *Appl. Surf. Sci.* 59, 299–303. doi: 10.1016/0169-4332(92)90131-G.

López-Aguado, C., Paniagua, M., Melero, J. A., Iglesias, J., Juárez, P., Granados, M. L., et al. (2020). Stable continuous production of γ-valerolactone from biomass-derived levulinic acid over zr–al-beta zeolite catalyst. *Catalysts* 10, 678. doi: 10.3390/catal10060678.

Morales, G., Melero, J. A., Iglesias, J., Paniagua, M., and López-Aguado, C. (2019). From levulinic acid biorefineries to γ-valerolactone (GVL) using a bi-functional Zr-Al-Beta catalyst. *React. Chem. Eng.* 4, 1834–1843. doi: 10.1039/c9re00117d.

Morin, S., Ayrault, P., Gnep, N. S., and Guisnet, M. (1998). Influence of the framework composition of commercial HFAU zeolites on their activity and selectivity in m-xylene transformation. *Appl. Catal. A: Gen.* 166, 281–292. doi: 10.1016/S0926-860X(97)00263-9.

Ostovar, S., Saravani, H., and Rodríguez-Padrón, D. (2021). Versatile functionalized mesoporous Zr/SBA-15 for catalytic transfer hydrogenation and oxidation reactions. *Renew. Energy* 178, 1070–1083. doi: 10.1016/j.renene.2021.06.095.

Sho, S. J., Kang, S. K., and Ryoo, R. (1999). PdPt bimetallic cluster supported on kl zeolite probed with xenon adsorption measurement, EXAFS/XANES and n-Butane hydrogenolysis. *Stud. Surf. Sci. Catal.* 121, 295–300. doi: https://doi.org/10.1016/S0167-2991(99)80082-2.

Simon, L. J., Van Ommen, J. G., Jentys, A., and Lercher, J. A. (2001). Sulfur-tolerant Pt-supported zeolite catalysts for benzene hydrogenation: I. Influence of the support. *J. Catal.* 201, 60–69. doi: 10.1006/jcat.2001.3236.

Talukdar, A. K., Bhattacharyya, K. G., and Sivasanker, S. (1993). Hydrogenation of phenol over supported platinum and palladium catalysts. *Appl. Catal. A: Gen.* 96, 229–239. doi: 10.1016/0926-860X(90)80012-4.

Tang, B., Li, S., Song, W. C., Yang, E. C., Zhao, X. J., Guan, N., et al. (2019). Hierarchical FAU-Type Hafnosilicate Zeolite as a Robust Lewis Acid Catalyst for Catalytic Transfer Hydrogenation. *ACS Sustain. Chem. Eng.* 7, 16329–16343. doi: 10.1021/acssuschemeng.9b03347.

Tangale, N. P., Niphadkar, P. S., Joshi, P. N., and Dhepe, P. L. (2019). Microporous and Mesoporous Materials Hierarchical K / LTL zeolite as solid base for aqueous phase hydrogenation of xylose to xylitol. *Microporous Mesoporous Mater.* 278, 70–80. doi: 10.1016/j.micromeso.2018.11.017.

Wang, W. J., Lin, H. Y., and Chen, Y. W. (2005). Carbon monoxide hydrogenation on cobalt/zeolite catalysts. *J. Porous Mater.* 12, 5–12. doi: 10.1007/s10934-005-5227-y.

Weitkamp, J., Karge, H. G., Pfeifer, H., and Holderich, W. (1994). *Zeolites and Related Microporous Materials: State of the Art*. 1st ed. , ed. J. Hölderich, W.; Karge, H. G.; Weitkamp Elsevier B.V.

Winoto, H. P., Ahn, B. S., and Jae, J. (2016). Production of g-valerolactone from furfural by a single-step process using Sn-Al-Beta zeolites: Optimizing the catalyst acid properties and process conditions. *J. Ind. Eng. Chem.* 40, 62–71. doi: https://doi.org/10.1016/j.jiec.2016.06.007.

Xu, S., Yu, D., Ye, T., and Tian, P. (2017). Catalytic transfer hydrogenation of levulinic acid to γ-valerolactone over a bifunctional tin catalyst. *RSC Adv.* 7, 1026–1031. doi: 10.1039/c6ra25594a.

Zhou, Y. H., Luo, Y. J., Lin, Y. T., and Huang, Y. B. (2018). Enhanced transfer hydrogenation activity of Zr-doped mesoporous silica through sol-gel method for the reduction of biomass-derived unsaturated carbon-oxygen bonds. *ChemistrySelect* 3, 11071–11080. doi: 10.1002/slct.201802176.
